# Experimentally and theoretically approaches for disperse red 60 dye adsorption on novel quaternary nanocomposites

**DOI:** 10.1038/s41598-021-89351-9

**Published:** 2021-05-11

**Authors:** N. K. Soliman, A. F. Moustafa, H. R. Abd El-Mageed, Omima F. Abdel-Gawad, Esraa T. Elkady, Sayed A. Ahmed, Hussein S. Mohamed

**Affiliations:** 1 Basic Science Department, Nahda University, Beni-Suef, Egypt; 2 Ministry of Health and Population, Central Administration of Environmental Affairs, Beni-Suef Branch, Beni-Suef, Beni-Suef Governorate Egypt; 3 Faculty of Science, Micro-Analysis and Environmental Research and Community Services Center, Beni-Suef University, Beni-Suef City, Egypt; 4 Chemistry Department, Faculty of Science, Beni-Suef University, Beni-Suef City, Egypt; 5 Research Institute of Medicinal and Aromatic Plants (RIMAP), Beni-Suef University, Beni-Suef City, Egypt

**Keywords:** Pollution remediation, Environmental sciences

## Abstract

A comprehensive study that combined both experimental and computational experiments was performed to evaluate the usage of organo-metal oxide nanocomposite for the elimination of disperse red 60 dye (DR) from aqueous solutions. Chitosan was modified by Schiff base to form nanoneedles chitosan-4-chloroacetophenone derivative. The derivatives were then impregnated with CeO_2_–CuO–Fe_2_O_3_ or CeO_2_–CuO–Al_2_O_3_ metal oxides to prepare a novel quarternary organo-metal oxide nanocomposite. The novel nanocomposite, chitosan-4-chloroacetophenone/CeO_2_–CuO–Fe_2_O_3_ (CF) and chitosan-4-chloroacetophenone/CeO_2_–CuO–Al_2_O_3_ (CA) are cheap and effective nano adsorbents that can be used for the uptake of DR from aqueous solution. The CF and CA nano-composites were characterized using different techniques. Moreover, the effect of adsorption parameters (initial DR concentration, time of contact, pH, temperature, and adsorbent mass) as well as CA and CF reusability tests were performed. Langmuir adsorption isotherm and pseudo-second-order kinetics models were best fitted with the adsorption process. The maximum amount of DR adsorbed was 100 mg/g on CF and CA at pH 2 and 4, respectively with a physical spontaneous, and exothermic adsorption process. Monte Carlo **(**MC) simulation studies indicated the adsorption of DR molecule on the CF and CA surfaces following a parallel mode in most of all studied configurations, confirming the strong interactions between the DR and surfaces atoms of CF and CA. The molecular structure analysis of DR dye adsorbed on the surface of CF and CA indicated that the adsorption process related to Van der Waals dispersion force. Consequently, this helps to trap DR dye molecules on the surface of CF and CA (i.e., physical adsorption), which supports our experimental results.

## Introduction

Environmental contaminants can begin to have toxic effects on individuals within animal or plant populations^1–5^. Great effort has been done to overcome the rapid growth of the wastewater problem, especially in industrial countries^6–11^. In terms of simplicity of usage and design, flexibility, cheapness, insensitivity to toxic pollutants, and safety, the adsorption technique for dye removal from wastewater effluent represents a wide area of interest^12–15^. In this regard, nanocomposite materials inter in a wide range of applications in numerous areas due to remarkable catalytic activity, high specific surface area high reducibility, and their chemical stability^16–19^. For wastewater treatment especially dyes removal, metal oxides, and metal oxides nanocomposite showed a high rate of dyes degradation and/or adsorption^20–26^. On the other hand, nanomaterials impregnated biomaterials represented new challenges as new adsorbents for dye removal from wastewater effluent^27–29^. After preparation of Fe_2_O_3_ impregnated biochar by pyrolysis of pulp and paper sludge at 750 °C, the obtained Fe_2_O_3_–biochar nanocomposite was used as a new adsorbent for methyl orange (MO)^27^ removal. The activated Fe_2_O_3_–biochar nano-composite showed high adsorption capacity than the unactivated biochar. This behavior is ascribed to the hybrid nature of the Fe_2_O_3_–biochar nano-composite and the adsorption expects to occur on biochar matrix and Fe_2_O_3_ nanocrystals sites. The adsorption process for the two catalysts follows pseudo-second-order kinetics. Also, the data of the adsorption process were best fit with the Freundlich isotherms with 20.53 mg/g maximum adsorption capacity. Another example is nano-ZnO/chitosan composite, which was fabricated and tested for the removal of Reactive Black 5. These nanocomposite showed high removal efficiency of 76% at optimum adsorption condition of pH 4 and an adsorbent weight of 0.2 g. The adsorption data were fit well with the Langmuir isotherm and represents a maximum adsorption capacity (qm) of 189.44 mg/g with a spontaneous and endothermic adsorption process^28^.

Giving the effectiveness of nanomaterials impregnated biomaterials, this study aims to prepare a novel quarternary nanocomposite CA and CF. The obtained new quarternary nanocomposites were used as an adsorbent for the DR dye from wastewater. In batch mode experiments, the effect of variables (e.g., starting DR concentrations, adsorbent doses, reaction times and temperatures, and pH values on DR dye elimination, adsorption kinetics and isotherms) were investigated. MC simulation was carried out to study the effect of modification of chitosan-4-chloroacetophenone Schiff base on the adsorption performance of DR dye onto Fe_2_O_3_ and Al_2_O_3_. We also studied the effect of increasing Fe_2_O_3_ and Al_2_O_3_ sizes on the adsorption energy and finally to discover the desorption sites of DR on CF and CA surface.

## Experiential details

### Raw materials, dyes, and reagents

Iron(III) nitrate was supplied from WINLAB, U.K. Cerium(III) sulfate was purchased from RIEDEL–DEHAEN AG company. Copper(II) sulfate was supplied from El-Gomhouria company, Egypt. Potassium hydroxide was supplied from international trade association company, Egypt. Bio basic Canada INC provided the chitosan with a degree of deacetylation 96% and MERCK-Schuchardt supplied 4-chloroacetophenone. Sigma Aldrich provided the DR dye, which was dissolved in distilled water. Sigma Aldrich supplied NaOH granules with 99.99% purity and 36% HCl, which were used to adjust the pH.

### Preparation of metal oxides nanocomposites

The CuO–CeO_2_ with a molar ratio of 5:95 was prepared by co-precipitation technology, in which KOH was used as a precipitating agent to co-precipitate copper(II) sulfate and cerium(III) sulfate at the prerequisite molar ratio. KOH was added dropwise to the precursor solution and sonicated for 30 min. Then the obtained precipitate was washed several times with distilled water and ethanol and dried at 105 °C for 24 h to calcine at 500 °C for about 3 h. The obtained nanosized CuO–CeO_2_ was supported on Al_2_O_3_ using wet impregnation technique^30,31^, where a small amount of water was added to a mixture of 40% weight percent of nano-sized CuO-CeO_2_ and 60% weight percent of Al_2_O_3_ powder and stirred at 60 °C for 1 h to form a paste and to attain a homogeneous impregnation of CuO–CeO_2_ in the surface of Al_2_O_3_ support. Finally, CuO–CeO_2_–Al_2_O_3_ impregnate was calcinated for 3 h at 400 °C in a box muffle furnace. CuO–CeO_2_–Fe_2_O_3_ with an equally molar ratio (1:1:1) was successfully prepared using the same previously described co-precipitation method.

**Scheme 1:**
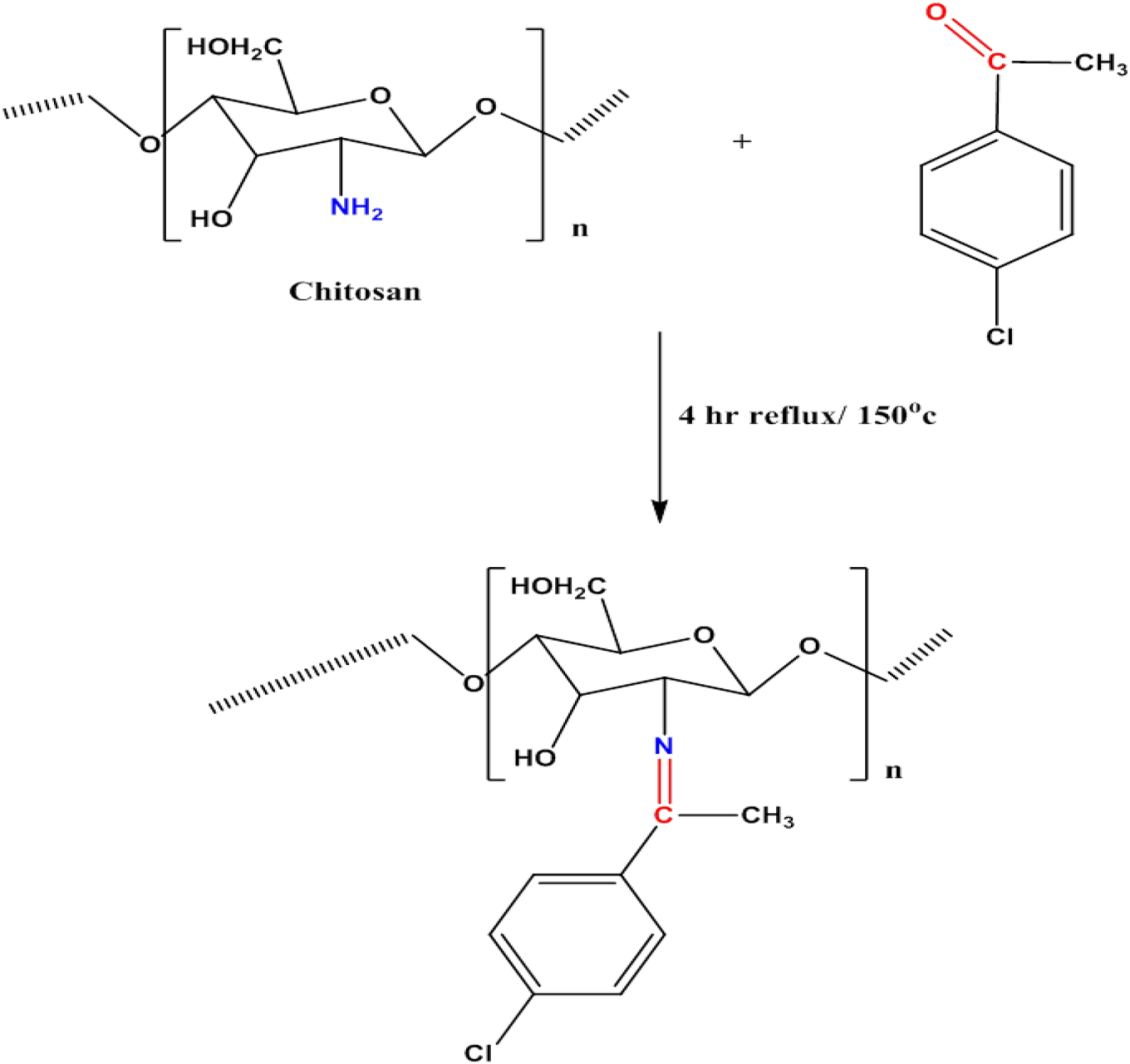
Schematic diagram of chitosan-4-chloroacetophenone Schiff base Synthesis.

Modified chitosan-4-Chloroacetophenone was synthesized by the addition of a pre-determined quantity of chitosan to 25 ml of 4-Chloroacetophenone. The mixture was refluxed at 150 °C for 4 h under stirring. To get rid of excess ketone, the newly obtained nano-organic material was filtered off and washed by distilled water and then dried at 60 °C. The following scheme (Scheme 1) represents the synthesis of chitosan-4-chloroacetophenone Schiff base.

Wet impregnation methodology was used to prepare the novel quarternary organo-metal oxides nano-composites, CA and CF. Equal weights of chitosan-4-chloroacetophenone and metal oxides were mixed and converted to paste by the addition of a small amount of distilled water and after that, the wet mixture was heated slowly under continuous stirring at 50 °C till complete vaporization of water. X-ray diffractometer (XRD), scanning electron microscope (SEM), Fourier transformer-infrared (FTI-R) spectrometer, transmission electron microscopic (TEM) and thermogravimetric analysis (TGA) were used to characterize the newly prepared CA and CF nano-composites. The TGA of the obtained samples was recorded by heating the samples from 10 to 600 °C with a rate of heating 10 °C/min under a flow of 25 ml of N_2_ gas/min*.*

### Samples characterizations

In the range from 4000 to 400 cm^−1^, FTIR spectrum was measured on SHIMADZU FTIR-8101 system (Shimadzu, Kyoto, Japan). Polystyrene film was used for the calibration of the frequency reading. The XRD analysis (JSX-60P JEOL diffractometer) was used for phase identification of the prepared nanomaterials. The TEM (2100 high-resolution TEM (JEOL Ltd, Tokyo, Japan)) analyses were used for the determination of surface morphology and particle size of the prepared nanomaterials. The adsorption process was followed up using UV/Vis spectrometer (Jasco V-350). The surface topography and morphology of the obtained nanomaterials were characterized using SEM (JEOL 5410, Japan) operating at 20 kV. The cross-sectional samples were prepared by fracturing the nanomaterials under liquid nitrogen, then the dried samples were coated by gold sputtering to provide electrical conductivity.

### Adsorption experiments

The DR is considered an anthraquinone dye with the molecular formula C_20_H_13_NO_4_. A 1000 mg/l stock solution was prepared by dissolving 1 g of DR dye in 1.0 L of distilled water^32^. The stock was then diluted with distilled water to prepare the required working solution concentrations. Using either a 0.1 M HCl solution or NaOH solution, the pH of all prepared solutions was adjusted to3, 5, 7, and 10.

All DR adsorption experiments were done in batch mode scale in various conditions including dye initial concentrations(10 – 100 mg /l), contact time (240 min), adsorbent dosage (0.05–0.2 g), pH (2–8), and temperature (20–80 °C) with continuous shaking. Four adsorption experiments series were implemented on CF and CA adsorbents at diverse adsorption circumstances, including initial dye concentration, adsorption temperature, adsorbents dosage, and initial pH of the solution as displayed in Table 1. The experiment time was set at 240 min and the volume of the solution was 50 ml in all experiments. The UV/Vis spectrophotometer was used to determine the variance in CR concentration by following the absorption peak. The reusability tests of CF and CA adsorbents were examined 4 times using 0.05 g of both adsorbents, 50 ml, 100 mg/l initial DR concentration for 240 min contact time at 20 °C and pH 6. CF and CA adsorbents were collected from the solution after each run, then washed with distilled water and set for the next run.

**Table 1:** Conditions of experimental tests.

Series	Dye concentration, mg/l	Adsorbent weight, g	Temperature, °C	pH value
1	10, 25, 50 and 100	0.05	20	6
2	100	0.05, 0.1, 0.15 and 0.2	20	6
3	100	0.05	20, 40, 60 and 80	6
4	100	0.05	20	2, 4, 6 and 8

The amount of DR uptake by the synthesized nanocomposite at equilibrium (qe(mg/g) and time t (qt), and the DR dye removal % were determined using Eqs. (1–3) respectively^8,33^:

1 $${\text{q}}_{{\text{e}}} = { }\left( {C_{o} - C_{e} } \right)\frac{V}{m}$$

2 $${\text{q}}_{{\text{t}}} = { }\left( {C_{o} - C_{t} } \right)\frac{V}{m}$$

3 $$DR \;dye \;removal{\text{ \% }} = { }\frac{{(C_{o} - C_{t} )}}{{C_{o} }} \times 100$$

where C_o_, C_t_, and C_e_ are the concentrations of DR in mg/l at the beginning, at time t, and at equilibration, respectively. V is the DR volume in mL and m is the CA and CF masses in mg. The presented results were the mean values of three independent experiments.

### Adsorption isotherm

Langmuir, Freundlich, and Tempkin isotherms have been applied to explain the adsorption isotherm of the fabricated nanocomposite, CF and CA, for the tested DR. The three models can be represented by Eqs. (4–6), respectively^34–37^:

4 $$\frac{{C_{e} }}{{q_{e} }} = \frac{1}{{K_{L} Q_{o} }} + \frac{{C_{e} }}{{Q_{o} }}$$

5 $$\log q_{e} = \log K_{F} + \frac{1}{n}\log C_{e}$$

6 $$q_{e} = {\text{B ln}}K_{T} + {\text{B ln}}C_{e}$$

where, Q_o_ = the maximum amount of dye removed by CA or CF nano-adsorbent (mg/g), K_L_ (L/mg), K_F_ (mg/g)_,_ and K_T_ (L/mole) denotes the Langmuir constant, Freundlich constant, and Temkin binding constant, respectively. The density of adsorption represents by n. B (J/mol) is a constant B = (RT/b) associated with the heat of adsorption. T (K^o^) is the absolute temperature. R represents the universal gas constant (8.314 J mol^−1^ K^−1^).

The value of the dimensionless separation factor (R_L_) based on Eq. (7) could be used to predict the degree of favorability of the Langmuir isotherm for equilibrium data^38^.

7 $$R_{L} = \frac{1}{{\left( {1 + K_{L} C_{max} } \right)}}$$

where C_max_ represents the maximum initial DR concentration.

### Adsorption kinetics

Different adsorption kinetics models such as Intraparticle diffusion, pseudo-first and second order in addition to the simple Elovich kinetic model were used for reviewing the kinetics models that best fit with the adsorption of DR onto CF and CA adsorbents.

Equations (8–11) represent the pseudo-first-order kinetics model, pseudo-second-order kinetics model, intraparticle diffusion model, and simple Elovich kinetic model, respectively^12–14,39–42^.

8 $${\text{ln }}\left( {{\text{q}}_{{\text{e}}} {-}{\text{ q}}_{{\text{t}}} } \right) \, = {\text{ lnq}}_{{\text{e}}} {-}{\text{ k}}_{{1}} {\text{t}}$$

9 $$\frac{t}{{q_{t} }} = \frac{1}{{k_{2} q_{e}^{2} }} + \frac{t}{{q_{e} }}$$

10 $$q_{t} = k_{3} t^{\frac{1}{2}} + I$$

11 $$q_{t} = \frac{1}{\beta }\ln \alpha \beta + \frac{1}{\beta }\ln t$$

where k_1_ (min^–1^) denotes the pseudo-first-order rate constant, k_2_ (g/mg min) is the pseudo-second-order rate constant and k_3_ symbolizes the intraparticle propagation rate constant. I is a constant and is associated with the boundary layer thickness. α implies the adsorption rate at time = 0 min (mg/min). β represents the surface coverage extent (g/mg).

### Thermodynamic study

The adsorption of DR was studied at various temperature 20, 40, 60 and 80 °C at pH of 6, 100 mg/l initial concentration of dye and CA and CF adsorbents dosage of 0.05 g per 50 ml of DR. Gibbs’ free energy change (ΔG) (J/mol), enthalpy change (ΔH) (J/mol) and ΔS (the change in entropy) (J/mol K) were calculated to recognize the adsorption behavior of DR. $\mathrm{\Delta G}$ could be calculated using Eq. (12)

12 $${\Delta G} = - {\text{RT }}\ln k_{c}$$

where $${k}_{c}=\frac{{q}_{e}}{{c}_{e}}$$

Vaŉt Hoff equation [Eq. (13)] can be used to calculate ΔH and ΔS using the slope and intercept obtained from the plot of $\mathrm{ln}{k}_{c}$ versus 1/T

13 $$\ln k_{c} = \frac{{{\Delta S}}}{R} - \frac{{{\Delta H}}}{RT}$$

### Computational details

#### Monte Carlo (MC) simulation

Firstly, We build Fe_2_O_3_ and Al_2_O_3_ simplex box nanocluster with different size (1,2, and 3 nm) by nanocluster builder implicit in Materials Studio software^43^, as follow: Fe_2_O_3_, 1 nm (X,Y, Z directions = 10 Å, Fe = 42, and O = 55 atoms), 2 nm (X,Y, Z directions = 20 Å, Fe = 316, and O = 495 atoms), and 3 nm (X,Y, Z directions = 30 Å, Fe = 1092, and O = 1543 atoms), Al_2_O_3_, 1 nm (X,Y, Z directions = 10 Å, Al = 60, and O = 76 atoms), 2 nm (X,Y, Z directions = 20 Å, Al = 430, and O = 563 atoms), and 3 nm (X, Y, Z directions = 30 Å, Al = 1287, and O = 1920 atoms). The interaction between chitosan-4-chloroacetophenone Schiff base with Fe_2_O_3_ and Al_2_O_3_ simplex box nanocluster with different sizes (1,2, and 3 nm) was studied by density functional theory (DFT) using the GGA-PBE (Generalized Gradient Approximation–Perdew Burke Ernzerhof) functional. The double numerical polarized (DNP) basis set was assigned. No spin-polarization effects were included in the exchange–correlation functional. The core electrons of all studied structures were preserved with the effective core potential and all electrons, respectively. These calculations were performed by DMol^3^ module^44,45^. To find the lowest structure configurations of chitosan-4-chloroacetophenone Schiff base modified Fe_2_O_3_ (CF) and chitosan-4-chloroacetophenone Schiff base modified Al_2_O_3_ (CA). Also, DR was optimized using DMol^3^ module at the same previous conditions. MC simulation was carried out in this work to study the effect of modification by chitosan-4-chloroacetophenone Schiff base on the adsorption of DR onto Fe_2_O_3_ and Al_2_O_3_ also, the effect of increasing the size of Fe_2_O_3_ and Al_2_O_3_ on the adsorption energy and to find the desorption sites of DR on CF and CA surface. MC simulation was carried out by the Adsorption Locator module in the Biovia Materials Studio software^46^ using the COMPASS force field (Condensed-phase Optimized Molecular Potentials for Atomistic Simulation Studies) as a force field and use current in the charges section. In the molecular dynamics (MD) simulations, the electrostatic and van der Waals terms were treated with Ewald and group-based methods, respectively. The MD was simulated under NPT ensemble for 4 ns, followed by isothermal–isobaric (NPT) conditions at 1 atm and 300 K for 6 ns, with a time step of 10 fs. The temperature and pressure were controlled by the Nose thermostat and Berendsen barostat, respectively. The velocity Verlet algorithm was used in the integration of the equations of motion^47^. The basic principles of MC simulation used in this work have been described by Frenkel and Smit^48^.

## Results and discussion

### Adsorbent characterizations

#### XRD characterizations

**Figure 1:**
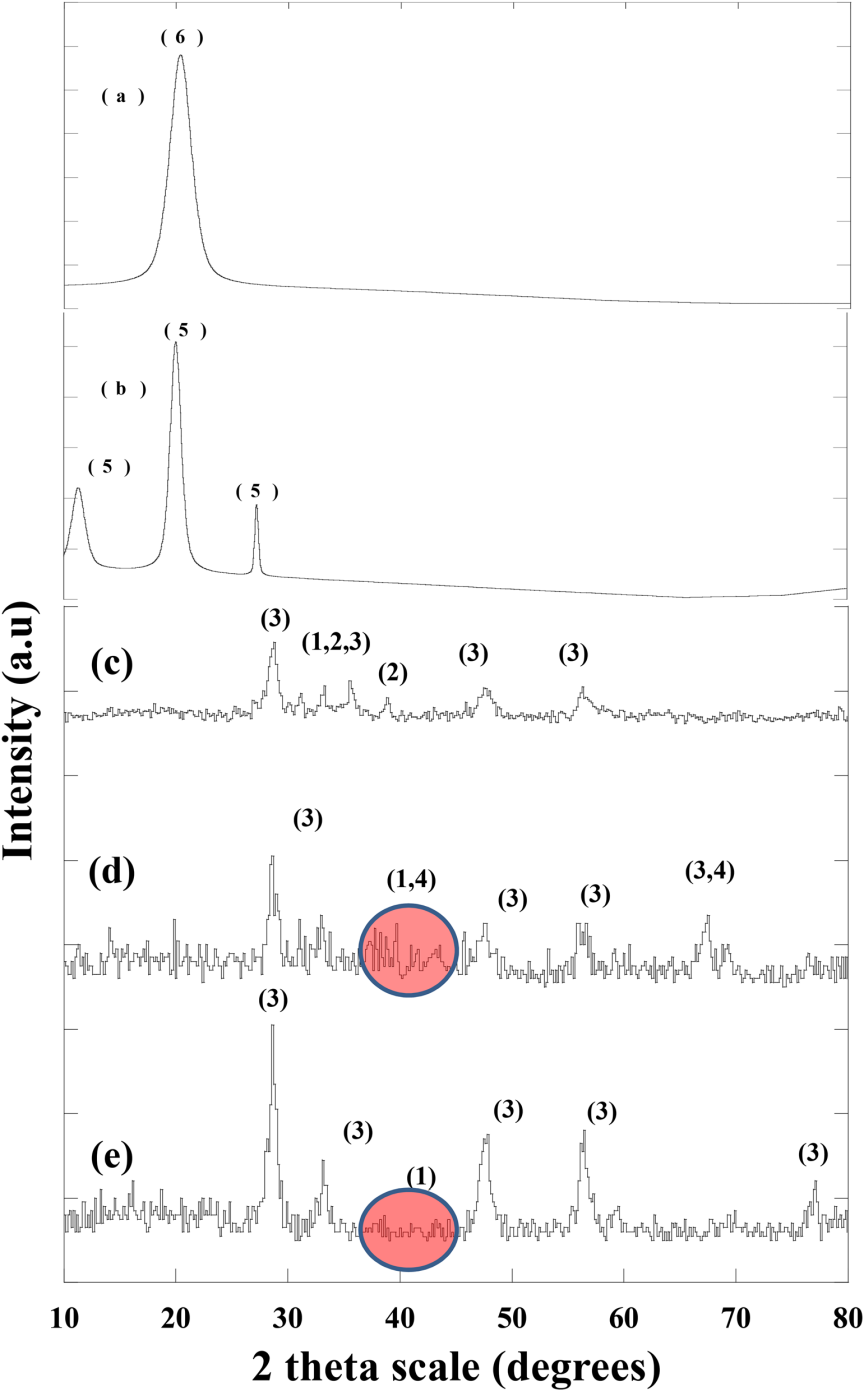
XRD pattern for: (**a**) chitosan-4-chloroacetophenone, (**b**) chitosan, (**c**) CuO–CeO_2_–Fe_2_O_3_, (**d**) CuO–CeO_2_–Al_2_O_3,_ and (**e**) CuO–CeO_2_, Where 1, 2, 3,4, 5 and 6 represent CuO, CeO_2_, Al_2_O_3_, Fe_2_O_3_, chitosan and chitosan-4-chloroacetophenone, respectively.

Figure 1 represents the XRD patterns of the prepared nano metal oxides and nanosized chloroacetophenone samples. Figure 1a,b showed the presence of CuO, CeO_2_ and Al_2_O_3_ nano metal oxides. The main peak of the three nano metal oxides was obtained at 29°, 39°, 40° for CeO_2_, CuO and Al_2_O_3_, respectively. The sharp peaks of CeO_2_ indicate its high crystallinity, on the other hand, the noise peaks of CuO and Al_2_O_3_ indicating their small crystal size and low crystallinity. We found no visible XRD peaks corresponding to copper oxides, which could designate that CuO was homogeneously dispersed into the CeO_2_ matrix. Otherwise, they were amorphous or undetectable by XRD^31^. The formation of nano-composites CeO_2_-CuO-Fe_2_O_3_, with main peaks at 29°, 39°, 40°, respectively were detected (Fig. 1a,b). The high and sharp peaks of the three metal oxides in the investigated nanocomposites reflected the high crystallinity of the three nano metal oxides compared to CuO–CeO_2_ and CuO–CeO_2_–Al_2_O_3_.

The calculated crystal size of the obtained metal oxides was found to be 11.7 and 7 nm for CeO_2_ and Al_2_O_3_, respectively. While CuO had undetected value in both CuO–CeO_2_–Al_2_O_3_ and CuO–CeO_2_, which may be interrelated to its small ratio, high dispersion on the surface of Al_2_O_3_ and very small crystal size. The small crystal size of CuO is reflected and confirmed by its noise peak and weak crystallinity. On the other hand, the calculated crystal size of the nano metal oxides in the nanocomposite CeO_2_–CuO–Fe_2_O_3_ was found to be 10.7, 35.5 and 13.1 nm, respectively. As deduce from peaks sharpness and high and the crystal size of the prepared nanocomposites, it may be deduced that the presence of Fe_2_O_3_ played a vital role in the crystal size growth and formation of CeO_2_–CuO–Fe_2_O_3_ nanocomposites.

XRD patterns of chitosan and its new nano-derivative (chitosan–4-chloroacetophenone) are presented in Fig. 1. The main peak of chitosan was displayed at 11° and 20° (Fig. 1). On the other hand, chitosan–4-Chloroacetophenone showed only one single peak at 20°. One of the two characteristic peaks of chitosan has been disappeared comparing with chitosan as shown in Fig. 1^49^.

#### TEM characterization

**Figure 2:**
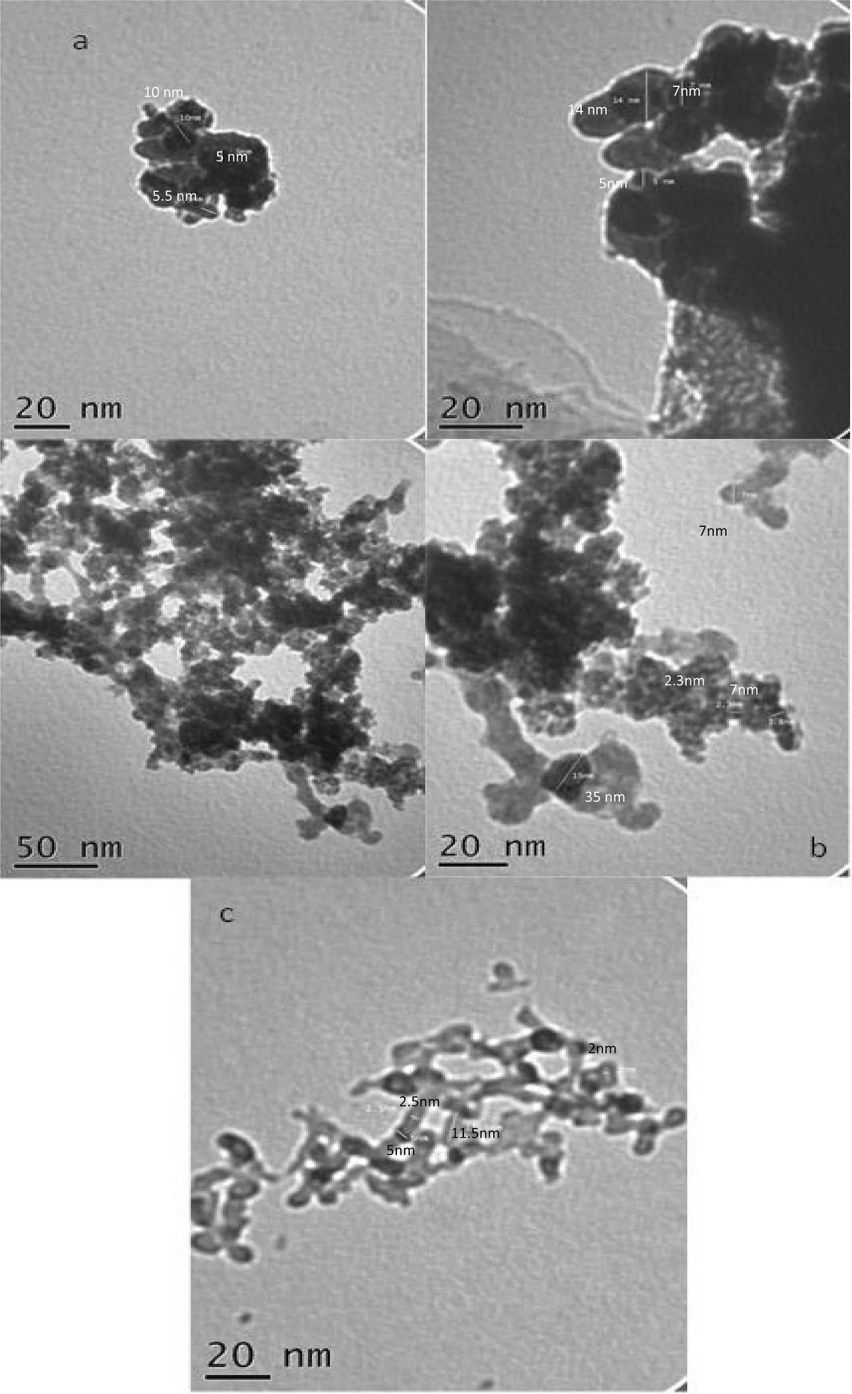
TEM micrographs of the prepared nanocomposites where (**a**) CuO–CeO_2_–Al_2_O_3_, (**b**) CuO–CeO_2_–Fe_2_O_3_, (**c**) CA.

TEM images of the investigated nanocomposites (Fig. 2) and the TEM image of CuO-CeO_2_-Al_2_O_3_ nanocomposite (Fig. 2a) were performed. The TEM images showed the existence of round agglomerated particles with diverse particles size. There are three particle sizes 5, 7 and 14 nm which reflect the existence of three metal oxides Al_2_O_3_, CuO, and CeO_2,_ respectively. The obtained particle sizes confirm and fit well with the XRD data.

Figure 2b showed TEM images of CuO–CeO_2_–Fe_2_O_3_ nanocomposite. The formation of rounded small particles with particle size 2.3, 3.5 and 7 nm which may be related to CeO_2_. On the other hand, there are particles with an undefined shape with a particle size of 15 nm in addition to the presence of a cylindrical shape with particle size 35–40 nm. The observed particle size also fitted well with the data obtained from XRD. Data in Fig. 2c represents the TEM image of CA, the image shows nanoparticles (5 nm) with round shape decorated nano-needles with 2.5 nm inner diameter and 11.5 nm particle size.

#### SEM characterization

**Figure 3:**
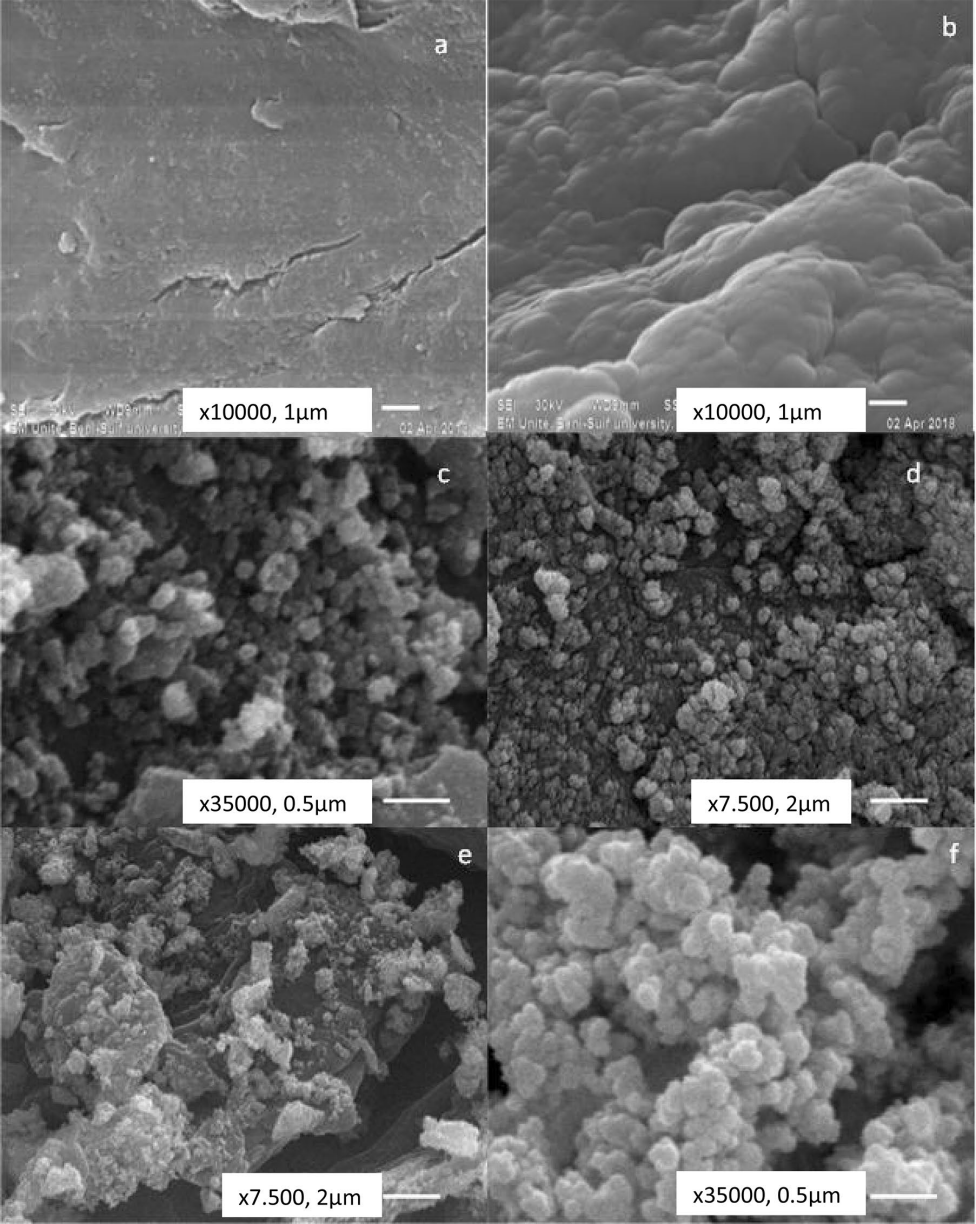
SEM micrographs of the prepared nanocomposites where (**a**) Chitosan, (**b**) Chitosan-4-chloroacetophenone, (**c**) CuO–CeO_2_–Al_2_O_3_, (**d**) CuO–CeO_2_–Fe_2_O_3_, (**e**) CA, (**f**) CF.

The surface morphology and topography of the chitosan, chitosan-4-chloroacetophenone, CuO–CeO_2_–Al_2_O_3_, CuO–CeO_2_–Fe_2_O_3_, CA and CF nanocomposites were illustrated (Fig. 3a–f). It was found that the surface of parent chitosan was fibrous and smooth with some cracks. The surface morphology of chitosan-4-chloroacetophenone differs from that of chitosan as a result of Schiff base reaction and this gives clear evidence for the Schiff base modification.

The SEM characterization of CuO–CeO_2_–Al_2_O_3_, CuO–CeO_2_–Fe_2_O_3_, CA and CF showed agglomerated rounded particles in microscale with a rough surface and deep hols. The impregnation process of the chitosan derivative presents a hard rough surface with huge cracks and edges (Fig. 3e,f).

#### FTIR spectrum

**Figure 4:**
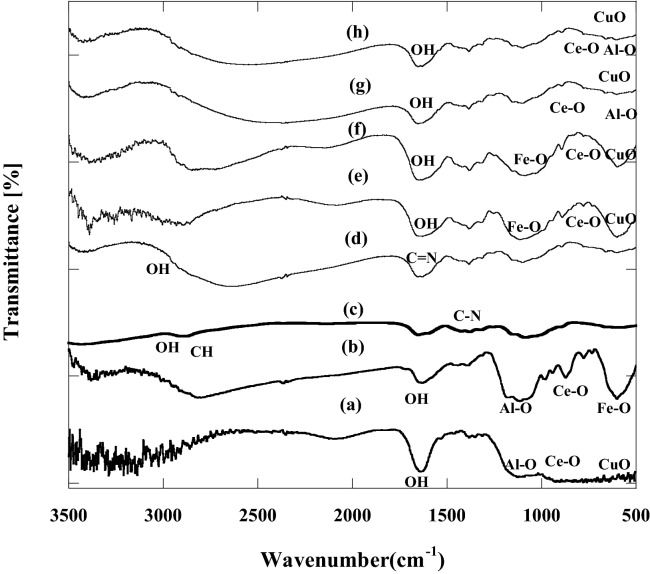
FT-IR before and after adsorption processes at 20 °C and pH 6 by 50 mg of: (a) CuO–CeO_2_–Al_2_O_3_ (b) CuO–CeO_2_–Fe_2_O_3_ (c) chitosan (d) Chitosan-4-chloroacetophenone (e) CF before adsorption (f) CF after adsorption (g) CA before adsorption (h) CA after adsorption.

Figure 4a,b and Supplementary Table S1 show the FTIR charts of CA and CF, respectively. The bands in the FT-IR spectrum which are displayed at 1648 and 1674 cm^−1^ for CA and CF, respectively, refer to O–H group bending vibration. The peak at 1097 cm^−1^ was associated with Al–O vibration mode, 598 cm^−1^ for Cu–O vibration mode and 425 cm^−1^ related to Fe–O vibration mode^50^. Ce–O showed its characteristic beak at 882 cm^−1^ in the case of CuO–CeO_2_–Fe_2_O_3_, which is shifted to 903 cm^−1^ in the case of CuO–CeO_2_–Al_2_O_3_. This shift may be attributed to the preparation technique (such as wet impregnation in the case of CuO–CeO_2_–Al_2_O_3_ and co-precipitation in the case of CuO–CeO_2_–Fe_2_O_3_) and/or the existence of Fe_2_O_3_^51^.

FTIR chart of chitosan was illustrated in Fig. 4c and Table S1, chitosan compound displayed four strong peaks at 1166, 1080, 1020 and 615 cm^−1^ which were representative of saccharide ring. The very strong broad peak at 3241 cm^−1^ should be allocated to the stretching vibration of OH groups, and intermolecular hydrogen bonds of polysaccharides and the extension vibration of NH_2_ groups. The representative peaks of primary amine of highly deacylated chitosan appear at 1651 cm^−1^ and 1407 cm^−1^ for amide I and amide II, respectively.

FTIR chart of chitosan 4-chloroacetophenone showed the presence of an O–H stretching vibration band between 3336 and 3026 cm^−1^ as found in Fig. 4d. The doublet peaks of –NH_2_ disappeared and two other peaks were detected to indicate the Schiff base reaction between C=O of acetophenones and –NH_2_ of chitosan to form N=C (imine group) which has a representative peak around 1690–1648 cm^−1^ ^52^. Also, the appearance of a new characteristic peak at 1380 and 1424 cm^−1^ for chitosan 4-chloroacetophenone confirms the presence of the aromatic ring.

Finally, the FT-IR spectrum of synthesized quaternary nanocomposites CA and CF was displayed (Fig. 4e,g). Generally, the IR spectrum shows that all peaks show no noticeable shift than that of metal oxides or chitosan 4-chloroacetophenone which means that CA and CF formed physically and there were no types of chemical reaction takes place between the nanometal oxides and chitosan 4-chloroacetophenone.

FTIR was also used to follow up the uptake of the DR dye onto the surfaces of the newly fabricated nanocomposites. Figure 4f,h represents FTIR of CF and CA, respectively, after the adsorption process. The shift in wavenumber confirmed the adsorption of DR dye on the surface of nanoadsorbent. Before adsorption, the nano-composites CF displayed three main peaks at 1631, 1080, 589 cm^−1^ (Fig. 4e). These peaks were shifted to 1640, 1045, 572 cm^−1^, respectively, after the adsorption process (Fig. 4f). On the other hand, before adsorption, CA nanocomposite exhibited three chief peaks at 1675, 1097, 649 cm^−1^ (Fig. 4 g) which are shifted to 1657, 1123, 598 cm^−1^, respectively, after the adsorption process (Fig. 4h).

#### TGA analyses

**Figure 5:**
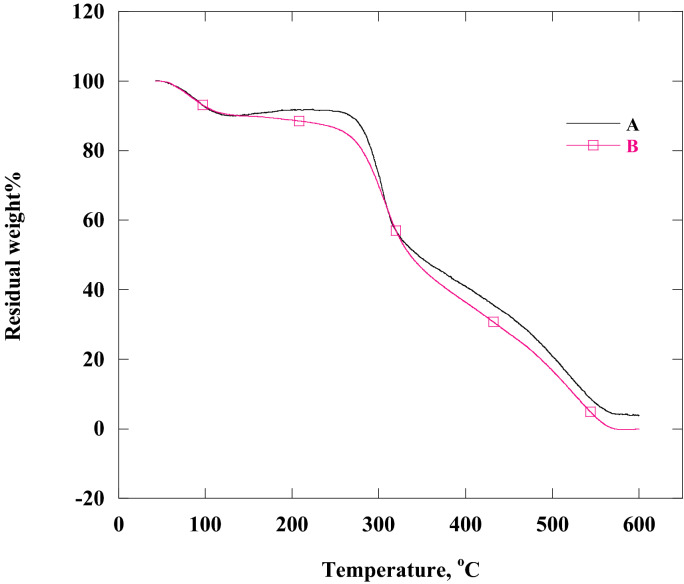
Thermal gravimetric analysis of (a) chitosan (b) Chitosan-4-ChloroacetoPhenone.

The TGA analyses of chitosan and chitosan-4-chloroacetophenone were showed in Fig. 5. In this data, chitosan-4-chloroacetophenone had thermal stability almost near to parent chitosan, but still less stable than chitosan. The low stability of the chitosan-4-chloroacetophenone compared with parent chitosan could be allied to the existence of the imine group (N=C) group in chitosan-4-chloroacetophenone, which was greatly influenced by heating than chitosan's amino group^53^.

Supplementary Table S2 showed weight loss % as a function of temperature. The first weight loss was observed around 100 °C which may be attributed to the evaporation of water molecules with a weight loss of about 10%. The 2nd step of weight loss was observed around 320 °C where the two nano organic compounds represented the same weight loss (approximately 47%) due to thermal decomposition. The 3rd step of weight loss was observed at 430 °C, where the parent chitosan showed 8% weight loss while its Schiff base derivative, chitosan-4-chloroacetophenone showed a high value of weight loss % (13%) due to its weak thermal stability compared to parent chitosan. The final weight loss step was observed at 565 °C, the parent chitosan still represents the low value of weight loss (30%) compared to its Schiff base derivative, chitosan-4-chloroacetophenone (30%).

### Factors influencing the adsorption process

#### Influence of initial DR concentration

**Figure 6:**
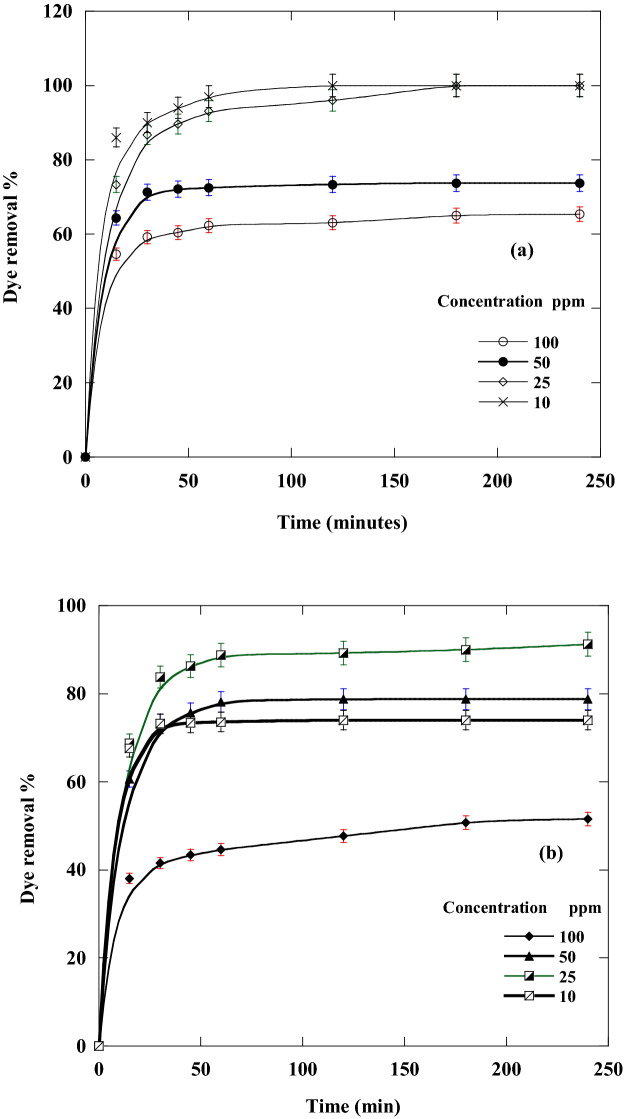
Effect of Disperse red 60 dye concentrations and contact time on the removal % of dye adsorbed at 20 °C and pH 6 by 50 mg of: (**a**) CF and (**b**) CA.

**Figure 7:**
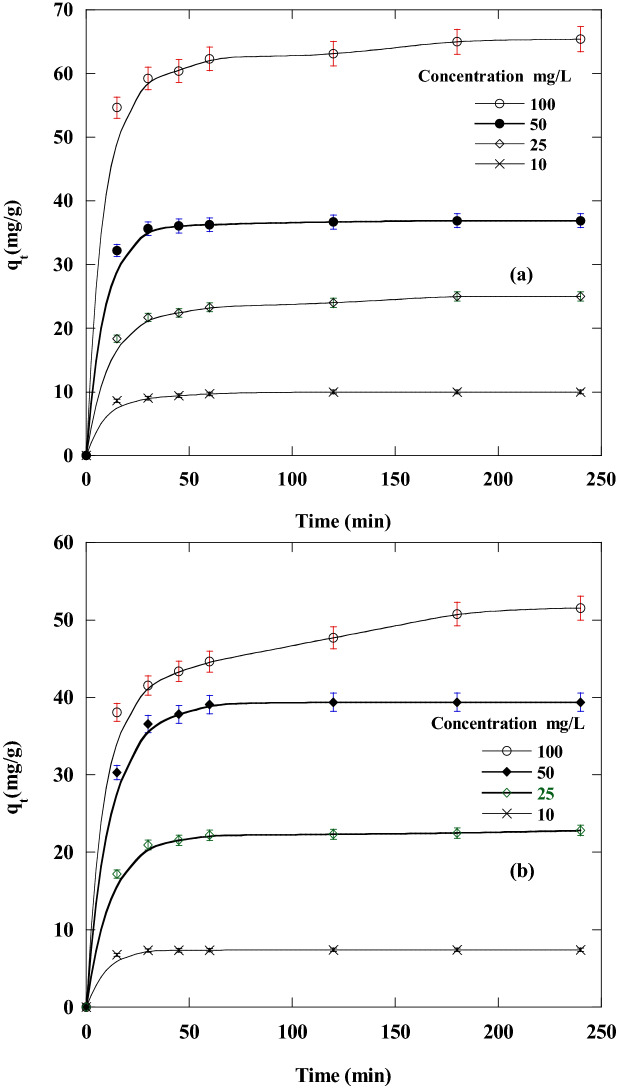
Effect of dye concentrations and contact time on the amount of dye adsorbed at 20 °C and pH 6 by 50 mg of new nano-biosorbents (**a**) CF and (**b**) CA.

The effect of contact time and initial concentrations of DR on the removal % and the amount of DR adsorbed using CA and CF was illustrated (Figs. 6 and 7), respectively. It was concluded that from the figure that during the first 45 min. In the first stage of the adsorption process, the adsorption capacity and the removal % of DR were very high and then they gradually decreased until they reached equilibrium. After reaching equilibrium, contact time had no discernible effect on the adsorption process using new sorbents.

CF nano-bio-adsorbent achieves 100% adsorption percentage for initial DR concentrations of 10 and 25 mg/l, while for the other initial concentrations, 50 and 100 mg/l, the adsorption percentage reached 73.75 and 65.38% respectively (Fig. 6a). On the other hand, for CA nano-adsorbent, DR removal percentage reached 51.5, 78.75, 91.25, and 74% for initial DR concentrations from 100 to 10 mg/l, in order (Fig. 6b). The lower DR removal % at lower initial DR concentrations may be accredited to the reduction in the concentration gradient with reducing initial DR concentration. Consequently, the draft forces, which can overawe the mass transfer resistance between DR adsorbate and CA and CF adsorbents will be decreased^12,54^.

The rapid removal rate at the initial stage of the adsorption progression could be allocated to the presence of an enormous number of available active adsorption spots on the CA and CF adsorbent's surface. The available hot sites will become fully occupied by the adsorbed DR molecules by increasing the time of contact between CA and CF adsorbent and DR adsorbate. As a result, repulsion forces were increased between DR molecules adsorbed on adsorbent surfaces and DR molecules in the bulk liquid phase^8^. For both nano-adsorbent CF and CA, the removal % of the investigated dye generally rises with decreasing the concentration of dye.

The quantities of DR adsorbed increase with the growth in the initial DR concentration. This could be accredited to the growth of the concentration gradient with rising the starting CR concentration (Fig. 7). Hence, appropriate growth in the draft forces occurs to overawed the mass transfer resistance between the DR adsorbate and CA and CF adsorbents^54^. The adsorption capacities of CF were found to be 9.82, 24.67, 36.9 and 65.2 (mg/g) for DR with initial concentrations of 10, 25, 50 and 100 mg/l, respectively. The adsorption capacities of CA were found to be7.4, 22.6, 39.4, and 51.1 (mg/g) for DR with initial concentrations 10, 25, 50 and 100 mg/l, respectively, at pH 6 and 20 °C.

#### Adsorbent dosage

**Figure 8:**
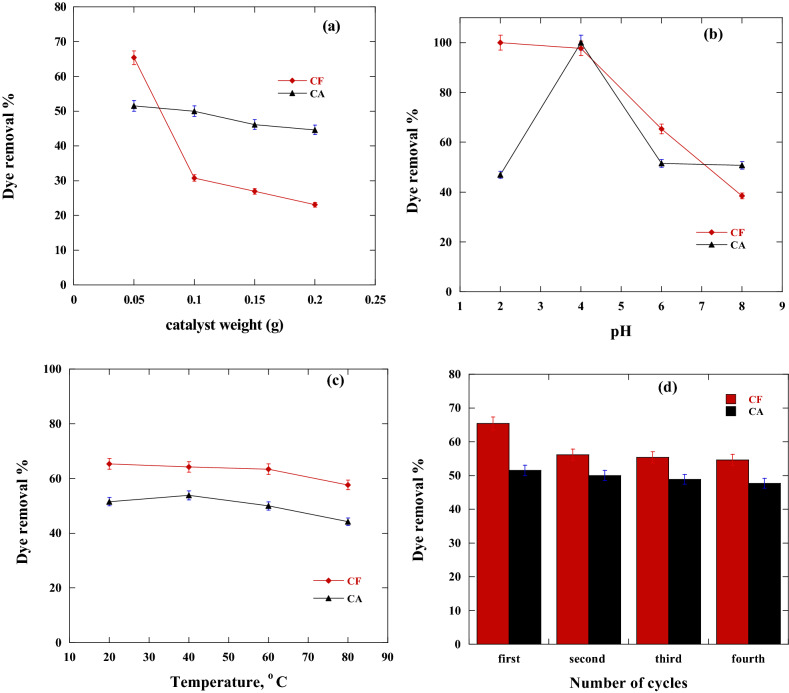
Effect of adsorption conditions on the removal % of DR dye by CA and CF where (**a**) represent the effect of adsorbent weight, (**b**) represent the effect of Initial pH of the solution, (**c**) represent the effect of adsorption temperature and (**d**) represent the reusability test.

The effect of CF and CA adsorbents dosage on the removal percentage of tested DR solution was examined to determine the optimal nanoadsorbent dosage that provides the best performance to determine the adsorption cost. Figure 8a illustrated graphically the change in the CR dye removal % as a function of adsorbent dosage. It was found that 0.05 g adsorbent per 50 ml of DR solution of an initial concentration of 100 mg/l was the best adsorbent dosage that gives the uppermost efficiency. Also, the removal % decreases from 65.38 to 23% and from 51.5 to 44.6% by increasing CF and CA dose from 0.05 to 0.2 g of adsorbent per 50 ml of DR, respectively, at a temperature of 20 °C and pH 6. This phenomenon can be explained by the formation of a dense screening layer at the nanoadsorbent surfaces as a result of adsorbent particle accumulation and a decrease in the distance between adsorbent molecules, a phenomenon known as the "screening effect" which occurs at higher adsorbent dosage. So, the condensed layer at the surface of the adsorbent hid the binding sites from DR molecules. Also, CA and CF overlapping resulted in a competition between DR molecules for restricted available binding sites. Aggregation or agglomeration at greater CA and CF doses increased the diffusion path length for DR adsorption causing a decrease in adsorption %^13,55–57^.

#### Influence of pH

pH has a great influence on the degree 0f dissociation and/or ionization of the DR molecules and the adsorbent surface. Consequently, the initial pH of the solution is a crucial player in the DR elimination by the CA and CF adsorbent^58^. The influence of the initial pH value on the removal % of DR by CF and CA nanocomposites was measured (Fig. 8b). Moreover, the pH effect was studied between pH 2 and pH 8 at an initial DR concentration of 100 mg/l and sorbent dosage of 0.05 g**.** The CF adsorbent shows removal percentages of 100, 97.6, 65.3 and 38.4 (%) for DR solution at pH of 2, 4, 6 and 8, in that order. The CA adsorbent showed removal percentages of 47, 100, 51.5 and 50.7 at pH of 2, 4, 6 and 8, respectively, at the same previously mentioned conditions. Generally, the DR removal % drops down with raising the pH of DR solution from pH2 to pH 8 except for CA at pH 2. The removal % is small because of the high H^+^ mobility and the protonation the adsorbent's surface. DR removal % decreases as a result of the competition between H^+^ ions and DR molecules during the adsorption process^59^. But at a higher pH value, H^+^ ions concentration decreases hence an elevation on the DR removal % by the CA adsorbent takes place^60^. For CF adsorbent, the high elimination % at low pH values may be owed to more + ve charge formation on the CF surface as a result of polar function groups protonation in acid medium. The DR anions strongly attracted to the positively charged adsorbent surface^61^. The declination in the DR removal % with the growth of pH might be owed to the deprotonation of the surface binding functional groups that revealed electrostatic repulsion force with the DR adsorbate anions. A high concentration of OH groups, at high pH, competes with DR molecules for CF adsorbent binding locations^62^. In other words, at elevated pH, the CF and CA surfaces were negatively charged, so the polarization of the electric double layer was reversed. Therefore, as shown in Fig. 8, the DR removal rate is reduced^63^.

#### Influence of temperature

The influence of temperature on the uptake % of DR onto CF and CA was done at different adsorption temperatures degrees. Figure 8c showed the influence of the temperature on the removal % of DR by CF and CA nanoadsorbents. The adsorption tests were done at 20, 40, 60 and 80 °C. For CF nanoadsorbent, the DR elimination % decrease from 65 to 57.7% with increasing temperature from 20 to 80 °C. This performance could be owed to the desorption behaviors of the adsorbed DR molecules at elevated temperatures. The temperature increases are responsible for the destruction of adsorption forces between hot binding spots of the nanoadsorbent and the DR adsorbate species, which may be due to the destruction of active sites, so, the best temperature for adsorption of DR onto CF is 20 °C^8^. The decrease in CR removal% with temperature increasing demonstrating that the adsorption process is exothermic.

For CA adsorbent, DR elimination % slightly increase with rising adsorption temperature from 20 to 40 °C, where it increased from 51.5 to 53.8% with changing temperature from 20 to 40 °C. The DR elimination % reduced from 53.8 to 44.2% by increasing temperature from 40 to 80 °C. With rising temperature from 20 to 40 °C, an increase in the DR removal% takes place due to the growth in the DR diffusion rate. By raising temperatures from 40 to 80 °C, a decrease in the DR elimination % occurred and this could be attributed to the desorption of DR molecules. DR molecules desorption resulted from the destruction of adsorption forces between hot binding spots of the nanoadsorbent and the DR adsorbate species. This was due to the destruction of active sites^64^, thus the best temperature for adsorption of DR onto CA was 40 °C.

#### Reusability of CF and CA

CF and CA reusability for the elimination of DR was followed four times with the same adsorbent and the same adsorbent dosage (Fig. 8d). The results showed that the removal strength of CA had not noticeably changed throughout the four adsorption cycles, while during the usage of CF adsorbent the DR removal % slightly decreased after the second cycle. For CF adsorbent, the documented dye removal % were 65.3%, 56.1%, 55.3% and 54.6% from the first to the fourth cycle in order. The reduction in the DR removal % could be ascribed to the agglomeration of the DR molecules onto the surface of CF. This consequently hind CF adsorbent surface and pores from the dissolved DR molecules, consequently a reduction in adsorption capacity take place^65^. For CA adsorbent, a slight decrease in the calculated DR removal % occurs where it changes from 51.5 to 50% and from 50 to 48.8% and from 48.8% to 47.6% for cycle 1 to cycle 4, respectively.

### Adsorption isotherm

The statistical significance of *R*^2^ (the correlation coefficient) for the linear plots *of C*e/*q*e versus *C*e, log(q_e_) versus log(C_e)_ and q_e_ versus Ln(C_e_) was used to fit the data to the Langmuir, Freundlich, and Tempkin isotherms, respectively.

**Figure 9:**
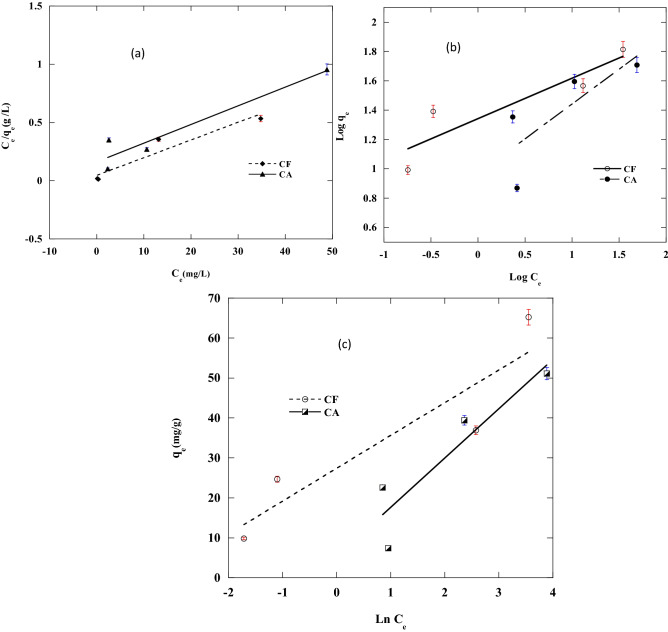
Adsorption isotherms for the adsorption of Disperse red 60 dye by 50 mg of CF and CA at 20 °C and pH 6: (**a**) Langmuir isotherm, (b) Freundlich isotherm, (**c**) Tempkin isotherm.

From the linear plots, the values of K_L_, K_F_, K_T_, Q_o_, n, B, and R^2^ were determined from Fig. 9 and recorded in Table 2. Our results demonstrate that DR adsorption on CF and CA adsorbents tracks the Langmuir isotherm models where the R^2^ value was the highest. i.e., the adsorption process almost tracks the Langmuir isotherm model. Therefore, the elimination of the dye happens at the active sites of the CA and CF nanoadsorbents on a mono surface layer, and the adsorbed DR molecules did not react with each other.

**Table 2:** Isotherm parameters for disperse red 60 adsorption on CF and CA.

Constants catalysts	Langmuir isotherm			
	Q _max_ (mg/g)	K_L_ (L/mg)	R_L_	R^2^
CF	65.9	0.326	0.03	0.9198
CA	62.3	0.099	0.09	0.9154

	Freundlich isotherm			
	n	K_F_	R^2^	
CF	3.6	21.8	0.8244	
CA	2.1	9.2	0.6272	

	Temkin isotherm			
	B (J/mol)	K_T_ (L/mole)	R^2^	
CF	8.2	28.3	0.8414	
CA	12.3	1.5	0.8460	

At 20 °C, the obtained R^2^ values calculated by the Langmuir isotherms of CF and CA adsorbents were 0.9198 and 0.9154, correspondingly. The value of R_L_ is < 1, signifying that the DR adsorption was favorable in the study case^66^.

### Adsorption kinetic models

**Figure 10:**
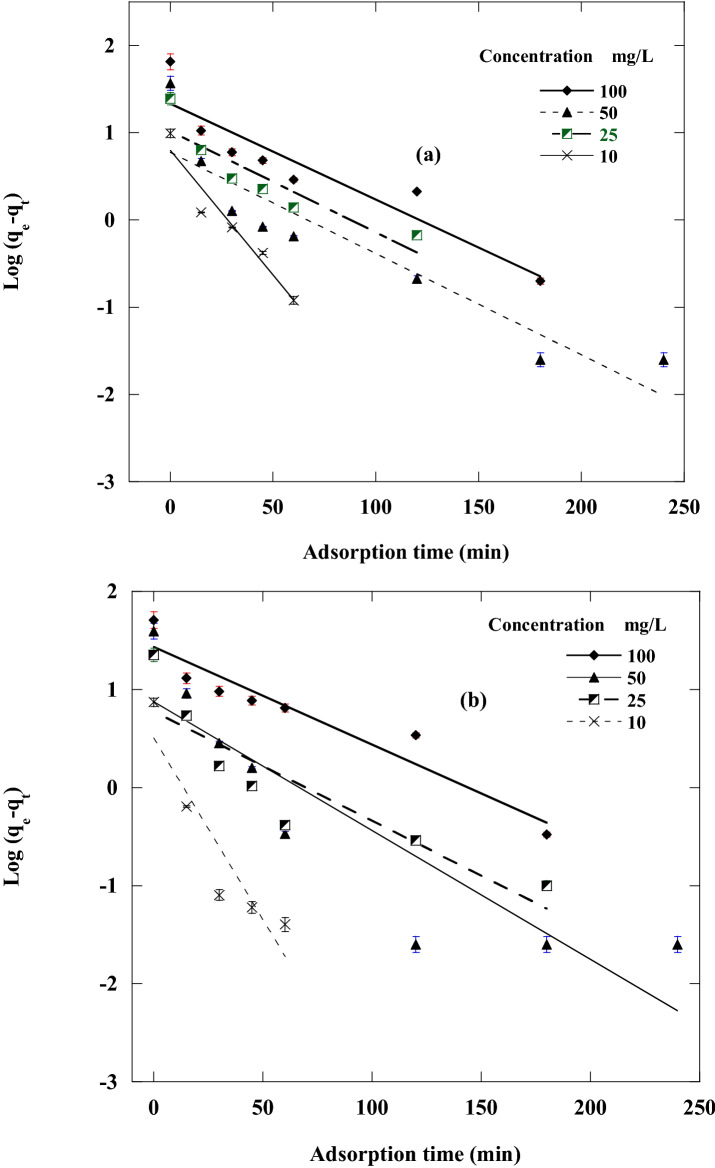
Pseudo-first-order sorption kinetics of Disperse red 60 dye at 20 °C and pH 6 by 50 mg of: (**a**) CF, (**b**)CA.

**Figure 11:**
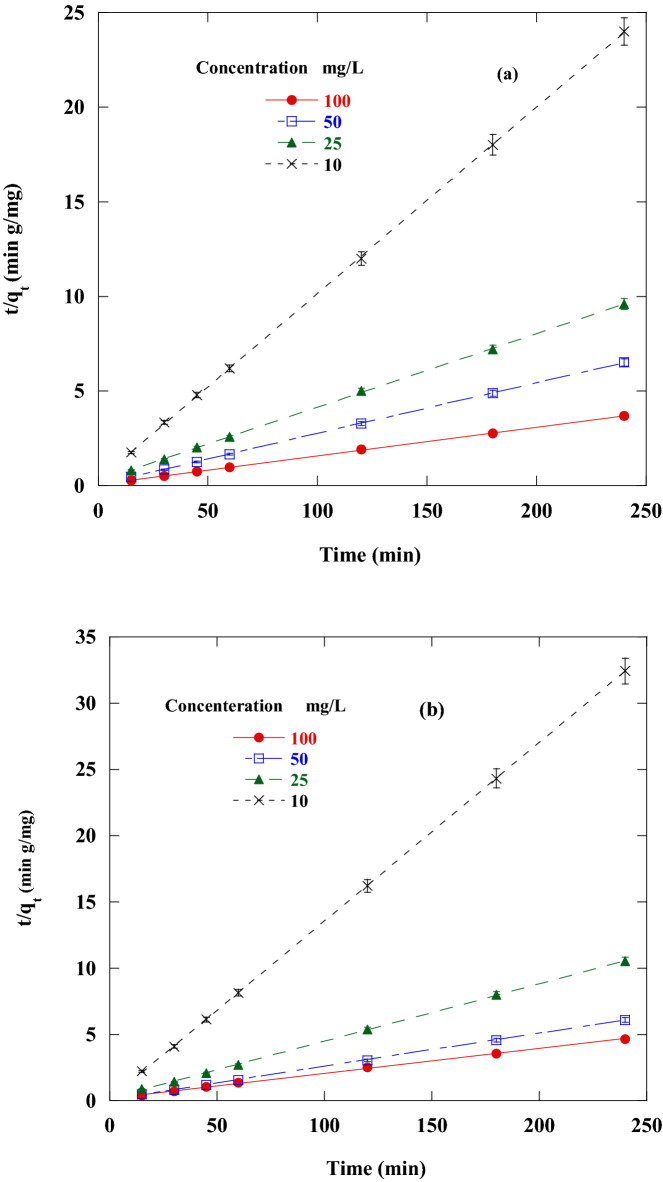
Pseudo-second-order sorption kinetics of Disperse red 60 dye at 20 °C and pH 6 by 50 mg of: (**a**) CF, (**b**) C.

**Figure 12:**
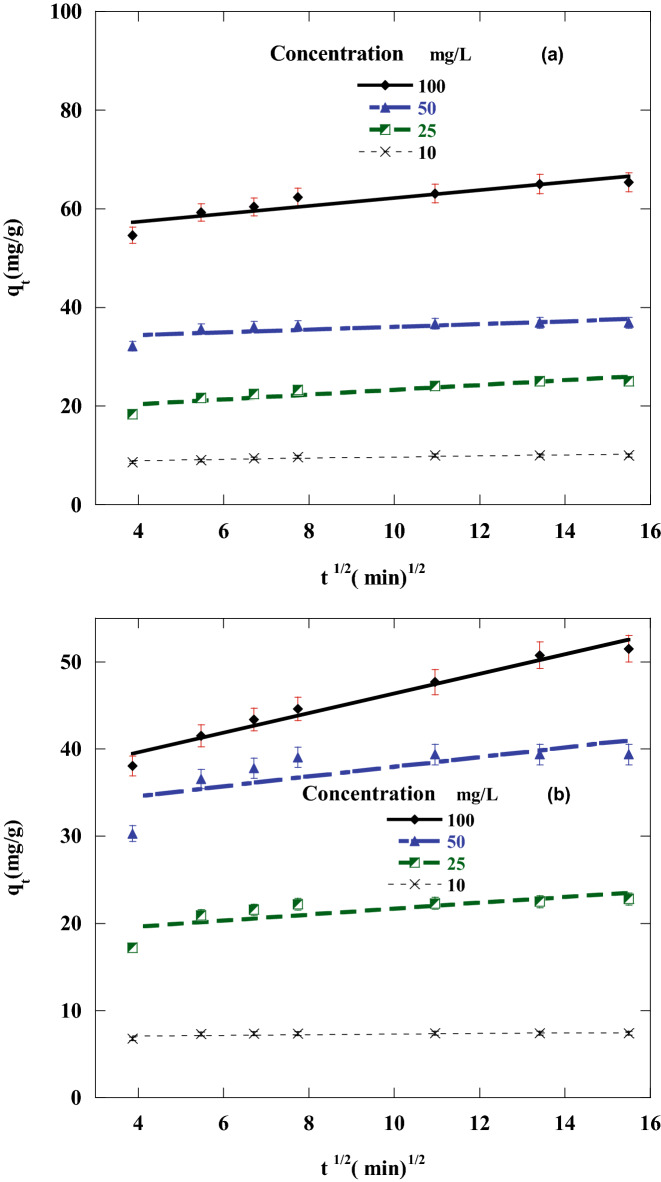
Plots for evaluating intraparticle diffusion rate constant for sorption of Disperse red 60 dye at 20 °C and pH 6 by 50 mg of : (**a**) CF, (**b**) CA.

**Figure 13:**
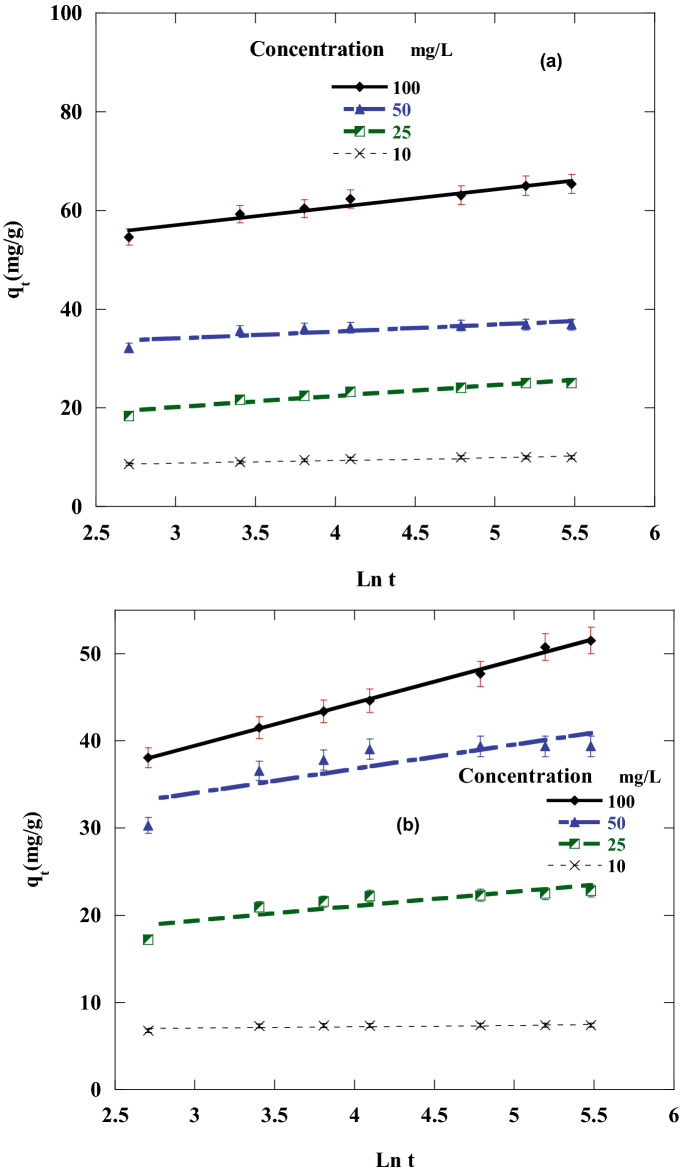
Plots for evaluating Elovich kinetic model for sorption of Disperse red 60 dye at 20 °C and pH 6 by 50 mg of: (**a**) CF, (**b**) CA.

To investigate the most appropriate adsorption kinetics model, the adsorption of DR on CA, and CF under various starting DR concentrations was measured. The first-order, second-order, intraparticle diffusion and Elovich kinetics linear graphs were represented in Figs. 10, 11, 12 and 13 by ploting ln (q_e_ – q_t_) versus t, $\frac{\mathrm{t}}{{\mathrm{q}}_{\mathrm{t}}}$ versus t, ${\mathrm{q}}_{\mathrm{t}}$ against ${\mathrm{t}}^\frac{1}{2}$ and q_t_ versus ln(t), individually. The adsorption kinetics parameters k_1_, k_2_, k_3_, q_e_, I, β, and α of the evaluation model in addition to R^2^ were calculated using the linear plots and depicted in Table 3.

**Table 3:** Parameters of the kinetic models for disperse red 60 adsorption on CF and CA.

Dye	CF				CA			
Concentration (mg/L)	100	50	25	10	100	50	25	10
**First order kinetic model**								
K_1_	11.03 × 10^–3^	11.02 × 10^–3^	11.62 × 10^–3^	28.59 × 10^–3^	9.9 × 10^–3^	13.1 × 10^–3^	11.1 × 10^–3^	37.1 × 10^–3^
Q_e_	3.66	2.17	2.77	2.22	4.19	2.39	2.17	1.65
R^2^	0.8632	0.8565	0.8173	0.9335	0.9104	0.8019	0.8018	0.8608
q_e_exp	65.2	36.9	24.67	9.82	51.1	39.4	22.6	7.4
**Second order kinetic model**								
K_2_	3.7 × 10^–3^	16.6 × 10^–3^	6.3 × 10^–3^	32.6 × 10 ^-3^	1.99 × 10^–3^	8.8 × 10^−3^	11.4 × 10^–3^	182.6 × 10^–3^
q_e_	66.3	37.1	25.6	10.1	53.2	40	23.3	7.4
R^2^	0.9998	0.9999	0.9998	0.9999	0.999	0.9998	0.9998	0.9999
q_e_ exp	65.2	36.9	24.67	9.82	51.1	39.4	22.6	7.4
**Intraparticle diffusion kinetic model**								
K_3_	0.799	0.283	0.481	0.115	1.12	0.55	0.33	0.034
I	54.1	33.2	18.4	8.4	35.1	32.2	18.3	6.9
R^2^	0.8408	0.5415	0.7883	0.7921	0.9686	0.5225	0.5662	0.4064
**Elovich kinetic model**								
α (mg/min)	13.5 × 10^5^	31.4 × 10^8^	950	5 × 10^5^	757	31.6 × 10^3^	95.7 × 10^2^	24.1 × 10^14^
β (g/mg)	0.27	0.72	0.45	1.88	0.204	0.362	0.603	5.7
R^2^	0.9418	0.7052	0.9104	0.9139	0.9954	0.6965	0.7286	0.5703

The linear fit and regression coefficient values in Table 3 for all the studied kinetic models confirmed that DR adsorption on CF and CA is well handled with the pseudo-second-order model with all DR concentrations. This was also confirmed from the good agreement between the values of calculated qe and the experimental qe Exp. There was an inverse relationship between the k_1_ value (pseudo-secondary rate constant) and the DR concentration tested. The same kinetics behavior has been observed also in different works^8,67,68^.

A straight line in the chart of *q*_*t*_ versus *t*^1/2^ proposes the applicability of the intraparticle diffusion model. *k*_2_ and *I* can be determined from the slope and intercept of the plot, respectively (Table 3). The R^2^ values (correlation coefficient) obtained from the model were relatively small and not satisfactory, also the value of *the intercept I* was not zero, demonstrating that the intraparticle diffusion model may not be the sole rate-controlling factor in determining the kinetics of the adsorption process. Compared to pseudo-first-order, Elovich kinetics model and intraparticle diffusion kinetic models, a respectable correlation coefficient was obtained for the pseudo-second-order kinetic model. This indicated that the adsorption of DR on the CA and CF follows pseudo-second-order rate model.

Our data reported that the pseudo-second-order kinetics model was dominant (Table 3). The pseudo-second-order kinetics model mechanism was implemented in two steps. The first stage, the external diffusion stage, included the mobility of DR molecules from all sides of the solution to the outer nanoadsorbent surfaces. The second step involved the adsorption and binding of DR molecules to the surfaces of CF and CA.

### Thermodynamic study

**Figure 14:**
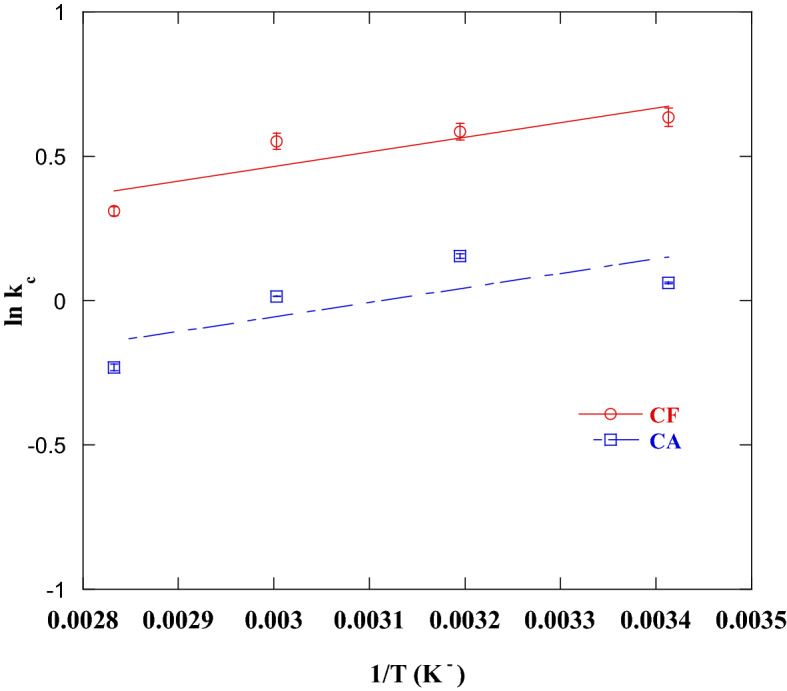
Van’t Hoff plot for Disperse red 60 dye adsorption onto CF and CA.

To determine the thermodynamic parameters, the adsorption of DR on CA, and CF under various temperature degrees. Table 4 shows the thermodynamic parameters obtained from linear plots of ln(kc) and 1/T (Fig. 14). The negative ΔG values of DR adsorption on the CF and CA adsorbents (except for the CA adsorbent at 353 K°) reveals the spontaneity of DR adsorption process. At elevated temperatures, the shift of ΔG value to a more + ve value indicated that the adsorption process was unfavorable at these temperatures. The ΔG value of the adsorption of DR on CF adsorbent is between − 0.91 and − 1.549 kJ/ mol, and the ΔG value of the adsorption of DR on CA adsorbent was in between − 0.035 and − 0.14 kJ/mol. All these values lied within the physical adsorption range of ΔG which extended from − 20–0 kJ/mol. This finding was also confirmed by the "n" value estimated from the Freundlich isotherm model, a value greater than unity refers to a physical adsorption process^69^. Also, the value of R_L_ is between 0 and 1, indicating that the adsorption of DR is favorable under experimental circumstances^66^. The negative ΔH value proves that the DR adsorption on CF and CA was an exothermic process. The –ve value of ΔS suggested a decrease in randomness at the solid/liquid interface due to DR adsorption on the surface of CF and CA^70^.

**Table 4:** Thermodynamic parameters for adsorption of disperse red 60 dye onto CF and CA.

Adsorbents	Temperature (K)	ΔG (J/mol)	ΔH (kJ/mol)	ΔS (J/mol. K)
CF	293	− 1549	− 4.236	− 8.813
	313	− 1523		
	333	− 1528		
	353	− 910		
CA	293	− 140	− 4.182	− 12.9
	313	− 350		
	333	− 35		
	353	526

### Comparison of CF and CA adsorption capacities with other adsorbents

The comparison between the adsorption capacity (qm) values of various adsorbents and CF and CA shows that there were significant differences between the qm values of different adsorbents^8,71^. For Moringa seed waste (MSW), its adsorption capacity was 196.8 mg/g^8^. For carboxymethyl cellulose (CMC), its adsorption capacity was 43.4 mg/g^71^. For free and fixed Lentinus concinnus fungal biomass, their adsorption amounts were 65.7 and 92.6 mg/g, respectively^71^. In our work, the maximum adsorption capacities of CF and CA were 100 mg/g at pH 2 and 4, respectively. This comparison showed that CF and CA show an average good ability to adsorb DR from an aqueous solution.

### MC simulation

**Figure 15:**
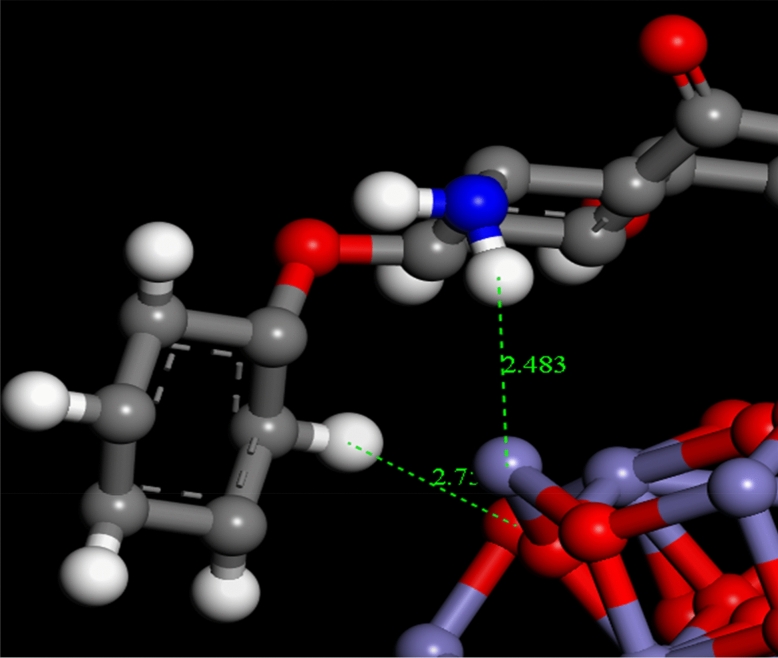
Snapshots for the adsorption configurations of DR adsorbed on CF-1 nm, the bond length is in Angstroms.

**Figure 16:**
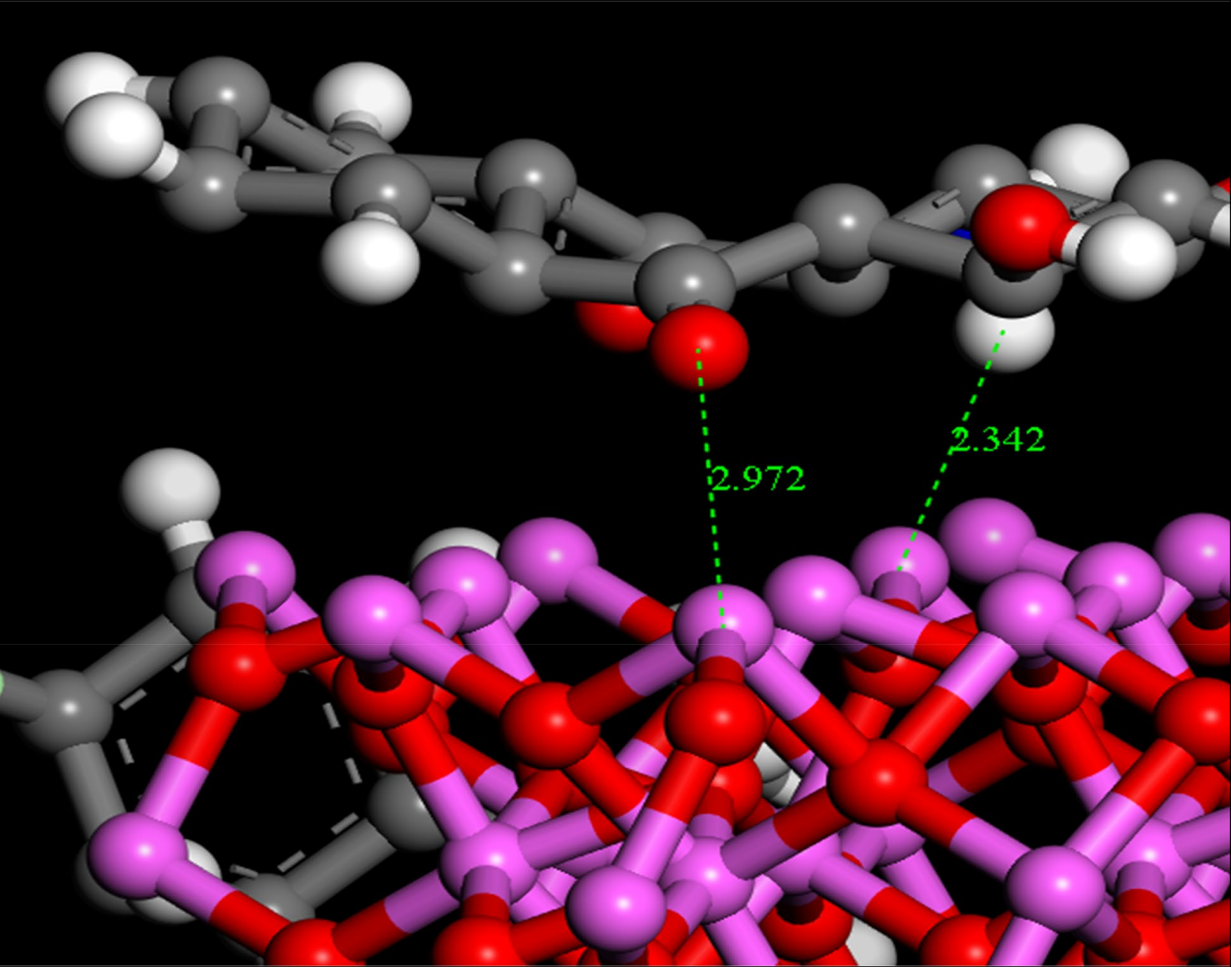
Snapshots for the adsorption configurations of DR adsorbed on CA-1 nm, the bond length is in Angstroms.

The lowest configurations were obtained due to the interactions between chitosan-4-chloroacetophenone Schiff base with Fe_2_O_3_ and Al_2_O_3_ (CF and CA) for a different size (1, 2, and 3 nm) in the dry system (no solvent) were summarized (Supplementary Figs. S1, and S2). The lowest adsorption configurations of the adsorption of DR on CF and CA surfaces for a different size in the dry system (no solvent) were also summarized (Supplementary Figs. S3 and S4). Adsorption energies for the adsorption configurations DR on CF and CA surfaces for a different size in the dry system (no solvent) were displayed in Table 5. Snapshots from the lowest configurations for adsorption configurations of DR adsorbed on CF and CA surfaces for 1 nm are displayed in Figs. 15 and 16 for clarity purposes and show the bond length formed between DR, CF and CA. DR molecule had diverse donor and acceptor sites for hydrogen bonds (HBs). Thus, it has formed numerous hydrogen bonds with the aluminum, iron, and oxygen atoms of CF and CA studied systems. As shown in Fig. 15, the hydrogen atoms of DR formed HBs and intramolecular HBs with the iron and oxygen atoms of the CF. Also, oxygen and hydrogen atoms of DR forms HBs and intramolecular HBs with the aluminum and oxygen atoms of the CA (Fig. 16). The adsorption (ΔE_ads_), interaction (E_int_), and deformation (E_def_) energies as well as substrate-adsorbate configurations (dE_ads_/d_Ni_)^72^, in which one of the adsorbate components has been removed, are summarized (Table 5). ΔE_ads_ for all configurations in this study are negative which revealed that the adsorption of DR molecule on CF and CA surfaces with different units was exothermic, energetically favorable and spontaneous, due to the existence of the intermolecular interactions. Also, increasing the CF and CA surface size significantly affects the adsorption energies for all configurations, in which the adsorption energies increasing with increasing the CF and CA surface size. Also, ΔE_ads_ of DR molecule absorbed on CF system was lower than those in the state of CA (Table 5). From MC simulation, it can be observed that the DR molecule adsorbs on the CF and CA surfaces following a parallel mode in most of all studied configurations, which confirms the strong interactions between the DR and CF and CA surfaces atoms. Analysis of the molecular structures of DR adsorbed on CF and CA surfaces show that the adsorption of DR onto chitosan-4-chloroacetophenone Schiff base surface may be related to the Van Der Waals dispersion forces, which can contribute to catching the DR molecules towards the CF and CA surfaces (physical adsorption) which confirm the results obtained in the experimental part.

**Table 5:** Adsorption energies (kcal/mol) for the adsorption configurations of DR adsorbed on chitosan-4-chloroacetophenone Schiff base, 5–10 units.

Systems	Adsorption energy	Rigid adsorption energy	Deformation energy	DR : dEad/dNi
DR-(CF − 1 nm)	− 30.7314	− 26.0254	− 28.7060	− 30.7314
DR-(CF − 2 nm)	− 32.5516	− 42.8785	− 27.6730	− 32.5516
DR-(CF − 3 nm)	− 33.5764	− 45.3713	− 28.2051	− 33.5764
DR-(CA − 1 nm)	− 33.4675	− 35.4561	− 28.0114	− 33.4675
DR-(CA − 2 nm)	− 35.4878	− 52.5294	− 27.9583	− 35.4878
DR-(CA − 3 nm)	− 37.7190	− 57.4152	− 27.3038	− 37.7190

### MD simulation

**Figure 17:**
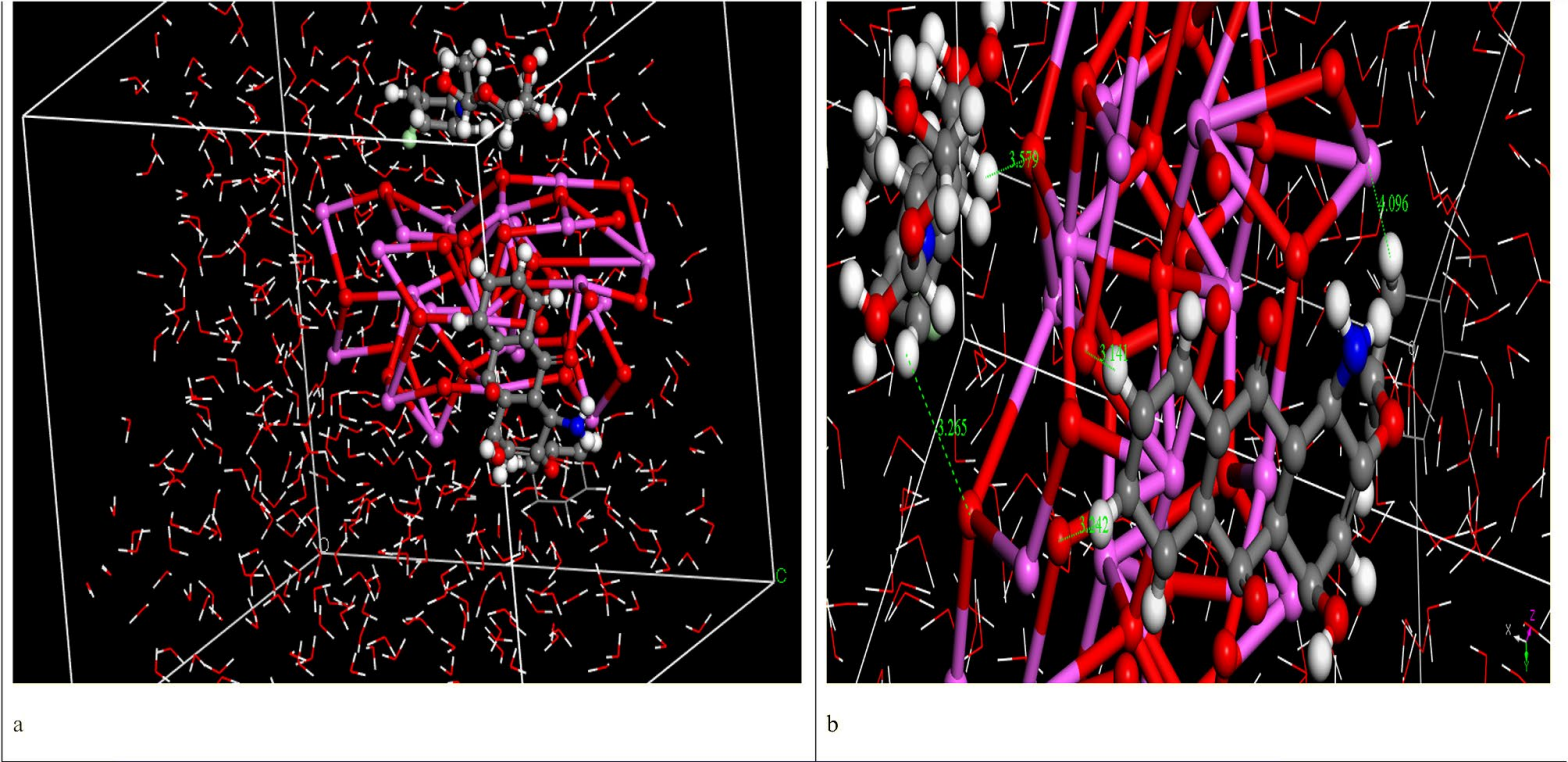
MD snapshots at 10 ns of the adsorption of DR molecule on the CA surface, the bond length is in Angstroms.

**Figure 18:**
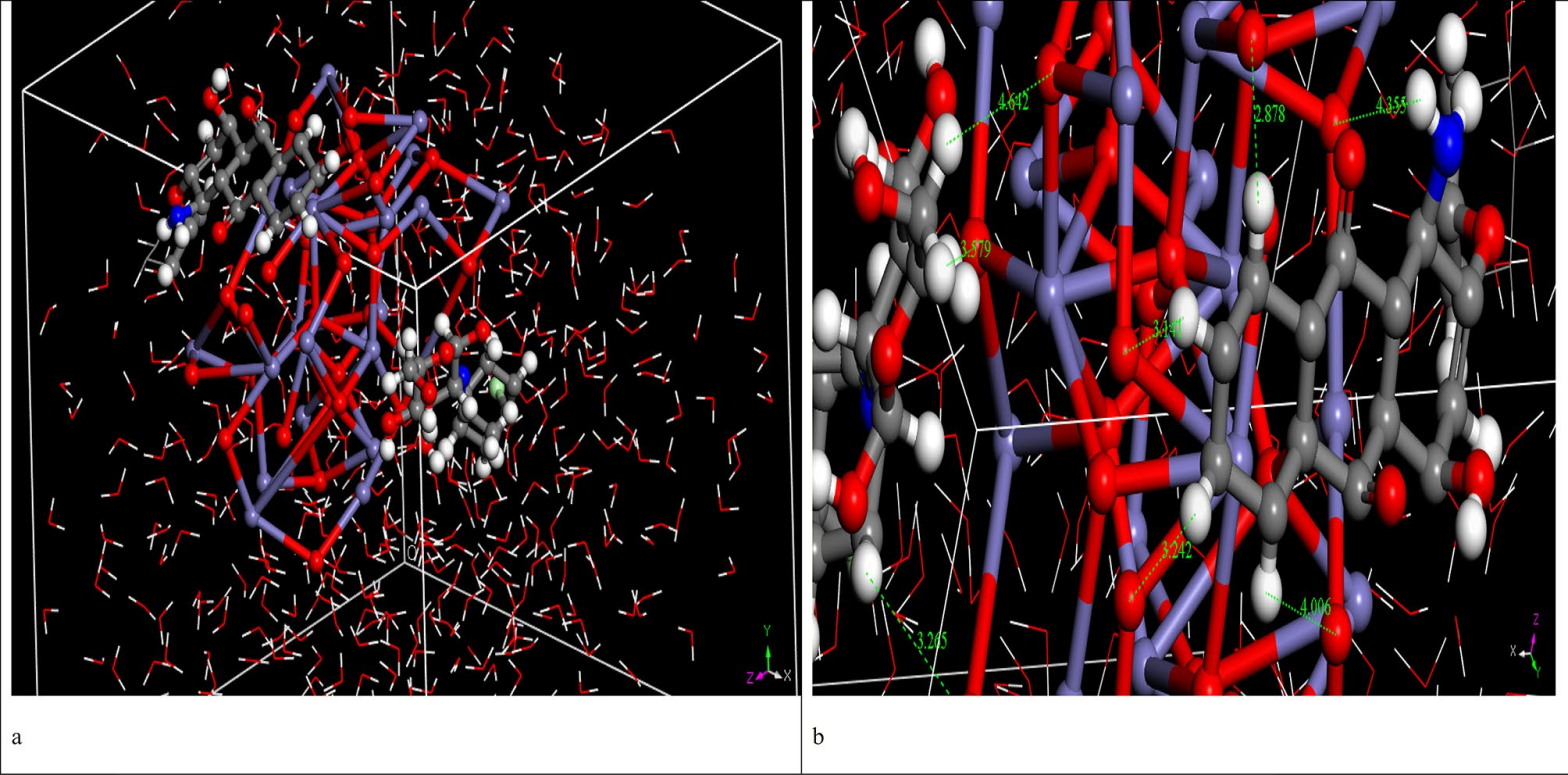
MD snapshots at 10 ns of the adsorption of DR molecule on the CF surface, the bond length is in Angstroms.

**Figure 19:**
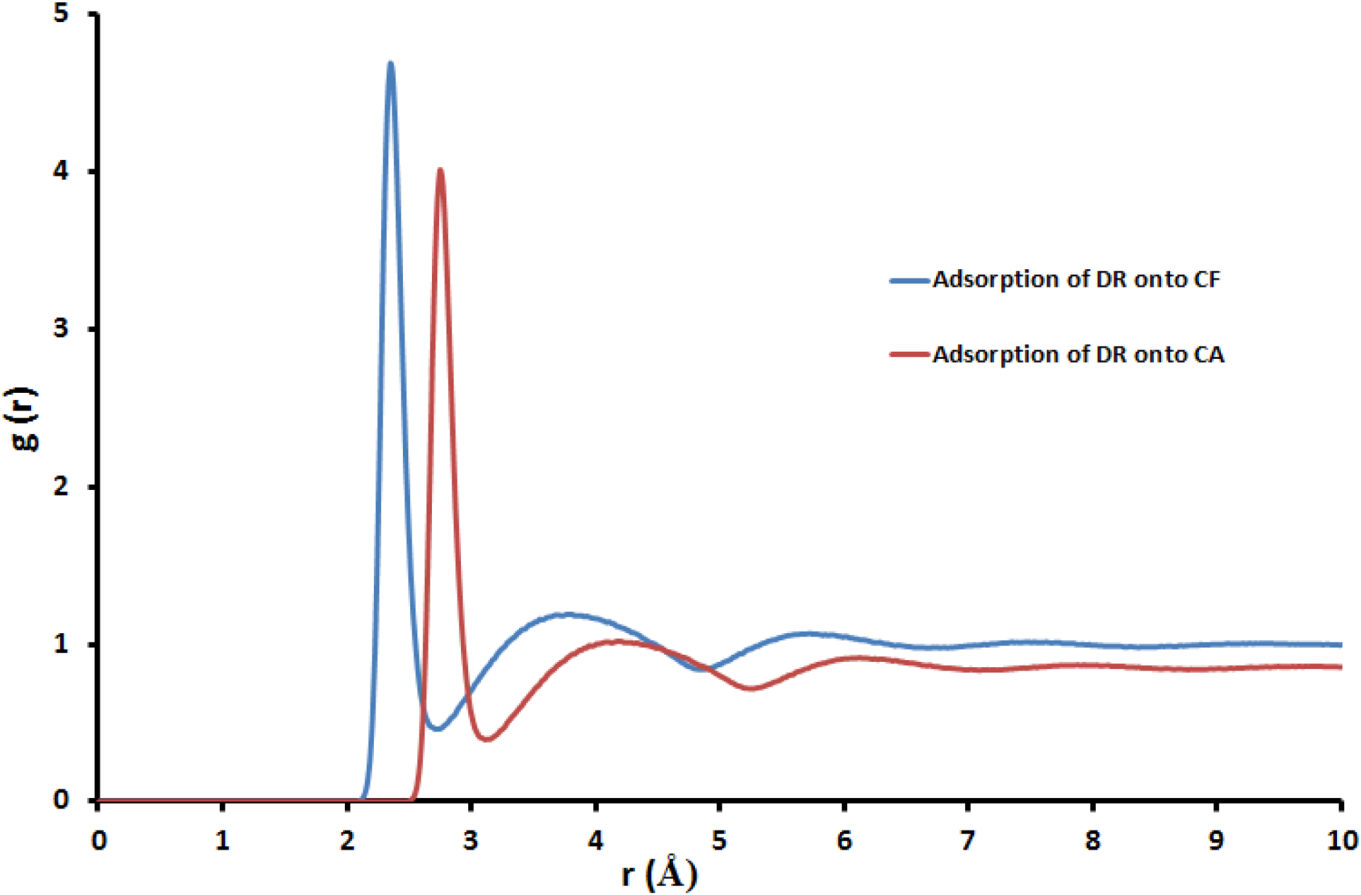
The RDFs for the interaction sites of *DR* molecule on CA and CF surface atoms in the presence of water at 10 ns.

The MD simulation was used to investigate the influence of the presence of solvent molecules (water) on the adsorption of DR on CA and CF surfaces, in which the MC lowest-energy structures (Fe_2_O_3_, and Al_2_O_3_ simple box nanoparticles at 1 nm) were simulated in explicit water using MD, each system was solvated in 500 water molecules. The ultimate simulation conformations of DR on CA and CF surface were shown in Figs. 17 and 18, respectively. The water molecules of the aqueous solution moved freely to interact with the DR atoms and the hydroxyl or oxygen atoms of CA and CF surfaces during the simulations. MD snapshots at 10 ns of the adsorption of DR on CA and CF surfaces are shown in Figs. 17a and 18a. The DR molecule has different hydrogen bond (HB) donor and acceptor sites, and thus, it has formed several hydrogen bonds with the hydroxyl groups of the CA and CF surfaces. The oxygen atoms of CA and CF were formed HBs with the hydroxyl hydrogen atoms of the DR molecule. Additionally, intramolecular HBs in DR were also formed through the hydroxyl hydrogen atoms with the hydroxyl oxygen atoms. Figures 17b and 18b displayed that DR molecules formed coordination bonds with CA and CF atoms in water. In the water system, intramolecular HBs between the functional groups of the DR molecule, as well as HBs between DR with water molecules, were observed. Thus, the MD simulation confirms that DR interacts with the CA and CF atoms even in presence of water molecules. Radial distribution function (RDF) was computed from the MD simulation to gain more insights into the stability of DR-CA and DR-CF complexes in water explicitly. This RDF can help us to understand the interaction between CA, CF and DR molecule. RDF was explained as the probability of locating particle “B” within the range (r + dr) of particle A, and is usually expressed as g(r). It was used to investigate the interaction between CA, CF and DR molecule, as well as describe the formation of hydrogen bonds with water. Figure 19 shows the RDFs obtained between the center of the mass of DR with CA and CF systems. The bonds formed between DR and CA have a bond length = 2.6 Å (Fig. 19). Also, the bonds formed between DR and CF have a bond length = 2.2 Å these two interactions take placed With high intensity confirmed that DR has strong interactions with CA and CF. RDFs reveal that DR adsorbs on CA and CF surface in the presence of water molecules.

## Conclusion

Wet impregnation technique was successfully used to prepare a novel quarternary organo-metal oxides nanocomposites CF and CA which were used as a new nano adsorbent for DR from an aqueous solution. The DR removal rate was high during the initial stage of the adsorption till the equilibrium state. The removal% generally improved with reducing the initial dye concentration where CF represent 100% removal at 10 and 25 mg/L while at 50 and 100 mg/L the removal % decreased to 73.75 and 65.38% respectively. The removal % reduced by increasing adsorbents dosage where it decreases from 65.38 to 23% and from 51.5 to 44.6% by increasing CF and CA dose from 0.05 to 0.2 g of adsorbent per 50 ml of DR, respectively, at 20 °C and pH 6. For the effect of operating temperature; the removal % decrease from 65 to 57.7% with rising temperature from 20 to 80 °C for CF nano adsorbent, however, for CA adsorbent the removal % decreased at an elevated temperature only. For the effect of changing pH of the solution; the removal % represent 100, 97.6% with changing the initial pH of the DR solution from 2 to 4 for CA and it decreases again to 65.3 and 38.4% with further increase in pH from 6 to 8 respectively, while CF nanocomposite shows removal % 100, 97.6, 65.3 and 38.4% at pH values of 2, 4, 6 and 8 respectively. The reducibility test for both adsorbents showed that CA was a good reusable adsorbent compared to CF for DR removal. The adsorption kinetics and isotherm of DR best fit with pseudo-second-order kinetics and Langmuir isotherms. The maximum adsorption capacities were 100 mg of DR/g of CF and CA at pH 2 and 4, respectively with is a physical spontaneous adsorption process. From MC simulations it was observed that ΔE_ads_ of DR molecule absorbed on CF system are lower than those in the state of CA which agree with the experimental data. Analysis of the molecular structures of DR adsorbed on CF and CA surfaces show that the adsorption may be related to the Van Der Waals dispersion forces (physical adsorption) which confirm the experimental data. MD simulation confirmed that DR adsorbs on CA and CF surface in the presence of water molecules.

## Supplementary Information

Supplementary Information.

## References

[1] Naudts K (2014). Future climate alleviates stress impact on grassland productivity through altered antioxidant capacity. Environ. Exp. Bot..

[2] Casasole G (2017). Neither artificial light at night, anthropogenic noise nor distance from roads are associated with oxidative status of nestlings in an urban population of songbirds. Comp. Biochem. Physiol. A.

[3] Zinta G (2018). Dynamics of metabolic responses to periods of combined heat and drought in *Arabidopsis thaliana* under ambient and elevated atmospheric CO2. J. Exp. Bot..

[4] Soliman N (2019). Factors affecting CO oxidation reaction over nanosized materials: A review. J. Market. Res..

[5] AbdElgawad H (2020). Maize roots and shoots show distinct profiles of oxidative stress and antioxidant defense under heavy metal toxicity. Environ. Pollut..

[6] Mohamed HS (2021). Adsorption of Mn+ 7 ions on chitosan/cellulose composite: Experimentally and theoretically approaches. J. Dispers. Sci. Technol..

[7] Mortada W, Moustafa A, Ismail A, Hassanien M, Aboud A (2015). Microwave assisted decoration of titanium oxide nanotubes with CuFe 2 O 4 quantum dots for solid phase extraction of uranium. RSC Adv..

[8] KhamisSoliman N, Moustafa AF, Aboud AA, Halim KSA (2019). Effective utilization of Moringa seeds waste as a new green environmental adsorbent for removal of industrial toxic dyes. J. Mater. Res. Technol..

[9] Gaafar AA, Ibrahim EA, Asker MS, Moustafa AF, Salama ZA (2016). Characterization of polyphenols, polysaccharides by HPLC and their antioxidant, antimicrobial and antiinflammatory activities of defatted moringa (*Moringa oleifera* L.) meal extract. Int. J. Pharm. Clin. Res..

[10] Rabie AM (2020). Instantaneous photocatalytic degradation of malachite green dye under visible light using novel green Co–ZnO/algae composites. Res. Chem. Intermed..

[11] Azzama E, Ahmedb SA, Mohamedb HH, Adlyb MA, Gada E (2019). Removal of iron(II) from wastewater in oil field using 3-(p-methyl) phenyl-5-thionyl-1, 2, 4-triazoline assembled on silver nanoparticles. Desalin. Water Treat..

[12] Soliman N (2019). Removal of chromium and cadmium ions from aqueous solution using residue of *Rumex dentatus* L. plant waste. Desalin. Water Treat..

[13] Mohamed HS (2019). Adsorption of Cd2+ and Cr3+ ions from aqueous solutions by using residue of Padina gymnospora waste as promising low-cost adsorbent. Heliyon.

[14] Soliman N (2019). Cd2+ and Cu2+ removal by the waste of the marine brown macroalga *Hydroclathrus clathratus*. Environ. Technol. Innov..

[15] Soliman NK, Moustafa AF (2020). Industrial solid waste for heavy metals adsorption features and challenges; A review. J. Market. Res..

[16] Bahgat M, Farghali AA, Moustafa AF, Khedr MH, Mohassab-Ahmed MY (2013). Electrical, magnetic, and corrosion resistance properties of TiO 2 nanotubes filled with NiFe 2 O 4 quantum dots and Ni–Fe nanoalloy. Appl. Nanosci..

[17] Abdallah H, Moustafa A, AlAnezi AA, El-Sayed H (2014). Performance of a newly developed titanium oxide nanotubes/polyethersulfone blend membrane for water desalination using vacuum membrane distillation. Desalination.

[18] Amin SK, Moustafa A, Aboud A, Abdallah H (2016). Catalyzation of esterification reaction using sulfated titanium dioxide nanotubes, experimental design and performance. Res. J. Pharm. Biol. Chem. Sci..

[19] Abo-Almaged H, Moustafa A, Ismail A, Amin S, Abadir M (2017). Hydrothermal treatment management of high alumina waste for synthesis of nanomaterials with new morphologies. Int. Ceram. Rev..

[20] Zayed M, Ahmed AM, Shaban M (2019). Synthesis and characterization of nanoporous ZnO and Pt/ZnO thin films for dye degradation and water splitting applications. Int. J. Hydrogen Energy.

[21] Farghali A, Khedr M, Moustafa A (2008). Photocatalytic activity and magnetic properties of nanocrystallite strontium hexaferrite prepared by self-flash combustion. Mater. Technol..

[22] Magdalane CM (2017). Evaluation on the heterostructured CeO2/Y2O3 binary metal oxide nanocomposites for UV/Vis light induced photocatalytic degradation of Rhodamine-B dye for textile engineering application. J. Alloy. Compd..

[23] Saikia P, Tmiah A, Das PP (2017). Highly efficient catalytic reductive degradation of various organic dyes by Au/CeO 2-TiO 2 nano-hybrid. J. Chem. Sci..

[24] Mohamed HS (2019). Nano metal oxide impregnated Chitosan-4-nitroacetophenone for industrial dye removal. Int. J. Environ. Anal. Chem..

[25] Shaban M, Ahmed AM, Shehata N, Betiha MA, Rabie AM (2019). Ni-doped and Ni/Cr co-doped TiO2 nanotubes for enhancement of photocatalytic degradation of methylene blue. J. Colloid Interface Sci..

[26] Shaban M, AbdAllah H, Said L, Ahmed AM (2019). Water desalination and dyes separation from industrial wastewater by PES/TiO2NTs mixed matrix membranes. J. Polym. Res..

[27] Chaukura N, Murimba EC, Gwenzi W (2017). Synthesis, characterisation and methyl orange adsorption capacity of ferric oxide–biochar nano-composites derived from pulp and paper sludge. Appl. Water Sci..

[28] Çınar S, Kaynar ÜH, Aydemir T, Kaynar SC, Ayvacıklı M (2017). An efficient removal of RB5 from aqueous solution by adsorption onto nano-ZnO/Chitosan composite beads. Int. J. Biol. Macromol..

[29] Ranjith KS, Manivel P, Rajendrakumar RT, Uyar T (2017). Multifunctional ZnO nanorod-reduced graphene oxide hybrids nanocomposites for effective water remediation: effective sunlight driven degradation of organic dyes and rapid heavy metal adsorption. Chem. Eng. J..

[30] Khedr M, Halim KA, Soliman N (2008). Effect of temperature on the kinetics of acetylene decomposition over reduced iron oxide catalyst for the production of carbon nanotubes. Appl. Surf. Sci..

[31] Khedra M, Nasrb M, Halimb KA, Farghalia A, Solimanc N (2014). Catalytic decomposition of hydrocarbon gas over various nanostructured metal oxides for hydrocarbon removal and production of carbon nanotubes. Int. J. Eng. Res. Gen. Sci..

[32] Bayramoğlu G, Ozalp VC, Arıca MY (2016). Removal of Disperse Red 60 dye from aqueous solution using free and composite fungal biomass of *Lentinus concinnus*. Water Sci. Technol..

[33] Khedr M, Halim KA, Soliman N (2009). Synthesis and photocatalytic activity of nano-sized iron oxides. Mater. Lett..

[34] Langmuir I (1918). The adsorption of gases on plane surfaces of glass, mica and platinum. J. Am. Chem. Soc..

[35] Freundlich H (1906). Over the adsorption in solution. J. Phys. Chem.

[36] Foo K, Hameed BH (2010). Insights into the modeling of adsorption isotherm systems. Chem. Eng. J..

[37] Temkin M, Pyzhev V (1940). Kinetics of ammonia synthesis on promoted iron catalysts. Acta Physiochim. URSS.

[38] Ozdemir O, Armagan B, Turan M, Celik MS (2004). Comparison of the adsorption characteristics of azo-reactive dyes on mezoporous minerals. Dyes Pigm..

[39] Xin N, Gu X, Wu H, Hu Y, Yang Z (2012). Application of genetic algorithm-support vector regression (GA-SVR) for quantitative analysis of herbal medicines. J. Chemom..

[40] Fan L, Luo C, Sun M, Qiu H, Li X (2013). Synthesis of magnetic β-cyclodextrin–chitosan/graphene oxide as nanoadsorbent and its application in dye adsorption and removal. Colloids Surf. B.

[41] Demiral H, Gündüzoğlu G (2010). Removal of nitrate from aqueous solutions by activated carbon prepared from sugar beet bagasse. Biores. Technol..

[42] Wu F-C, Tseng R-L, Juang R-S (2009). Initial behavior of intraparticle diffusion model used in the description of adsorption kinetics. Chem. Eng. J..

[43] Accelrys. (Accelrys Software, Inc., 2006).

[44] Delley B (1990). An all-electron numerical method for solving the local density functional for polyatomic molecules. J. Chem. Phys..

[45] Delley B (2000). From molecules to solids with the DMol 3 approach. J. Chem. Phys..

[46] Uzunova EL, Mikosch H (2013). Adsorption and activation of ethene in transition metal exchanged zeolite clinoptilolite: A density functional study. ACS Catal..

[47] Abdelrheem DA (2020). Bis-indole alkaloid caulerpin from a new source *Sargassum platycarpum*: isolation, characterization, in vitro anticancer activity, binding with nucleobases by DFT calculations and MD simulation. J. Biomol. Struct. Dyn..

[48] Frenkel, D. & Smit, B. Vol. 1 1–638 (Elsevier (formerly published by Academic Press), 2002).

[49] Sousa KS, SilvaFilho EC, Airoldi C (2009). Ethylenesulfide as a useful agent for incorporation into the biopolymer chitosan in a solvent-free reaction for use in cation removal. Carbohydr. Res..

[50] Masoudi RMH, Azin E, Taheri RA (2018). Adsorption of cadmium from aqueous solutions by novel Fe3O4-newly isolated Actinomucor sp. bio-nanoadsorbent: Functional group study. Artif. Cells Nanomed. Biotechnol..

[51] De Marzi LMA, De Lapuente J, Ramos D, Borras M, Di Gioacchino M, Santucci S, Poma A (2013). Cytotoxicity and genotoxicity of ceria nanoparticles on different cell lines in vitro. Int. J. Mol. Sci..

[52] Mohamed A, Sabaa W, El-Ghandour A, Abel-Aziz M, Abdel-Gawad O (2013). Preparation, characterization and antimicrobial activity of carboxymethyl chitosan schiff bases with different benzaldehyde derivatives. J. Am. Sci..

[53] Tirkistani FA (1998). Thermal analysis of some chitosan Schiff bases. Polym. Degrad. Stab..

[54] Sharma YC (2009). Optimization of parameters for adsorption of methylene blue on a low-cost activated carbon. J. Chem. Eng. Data.

[55] Tahir MAB, Iqbal M (2016). Solar red and brittle blue direct dyes adsorption onto *Eucalyptus Angophoroides* bark: Equilibrium, kinetics and thermodynamic studies. J. Environ. Chem. Eng..

[56] Adeela Kanwal HNB, Iqbal M, Noreen S (2017). Basic dye adsorption onto clay/MnFe2O4 composite: A mechanistic study. Water Environ. Res..

[57] Pons MP, Fuste MC (1993). Uranium uptake by immobilized cells of *Pseudomonas* strain EPS 5028. Appl. Microbiol. Biotechnol..

[58] Mohan SV, Rao NC, Karthikeyan J (2002). Adsorptive removal of direct azo dye from aqueous phase onto coal based sorbents: A kinetic and mechanistic study. J. Hazard. Mater..

[59] Foo K, Hameed B (2012). Preparation, characterization and evaluation of adsorptive properties of orange peel based activated carbon via microwave induced K 2 CO 3 activation. Biores. Technol..

[60] Sprynskyy M, Buszewski B, Terzyk AP, Namieśnik J (2006). Study of the selection mechanism of heavy metal (Pb 2+, Cu 2+, Ni 2+, and Cd 2+) adsorption on clinoptilolite. J. Colloid Interface Sci..

[61] Ansari RMZ (2010). Removal of eosiny, an anionic dye, from aqueous solutionsusing conducting electroactive polymers. Iran. Poly. J..

[62] Heibati BR-CS, Al-Ghouti MA, Asif M, Tyagi I, Agarwal S, Gupta VK (2015). Kinetics and thermodynamics of enhanced adsorption of the dye ar 18 using activated carbons prepared from walnut and poplar woods. J. Mol. Liq..

[63] Elkady M, Hussein M, Salama M (2015). Synthesis and characterization of nano-activated carbon from el maghara coal, Sinai, Egypt to be utilized for wastewater purification. Am. J. Appl. Chem..

[64] SaimaNoreen HNB, Zuber M, Zahid M, Asgher M (2017). Removal of actacid orange-RL dye using biocomposites: Modeling studies. Pol. J. Environ. Stud..

[65] Naghizadeh A (2016). Regeneration of carbon nanotubes exhausted with humic acid using electro-Fenton technology. Arab. J. Sci. Eng..

[66] Hall KR, Eagleton LC, Acrivos A, Vermeulen T (1966). Pore-and solid-diffusion kinetics in fixed-bed adsorption under constant-pattern conditions. Ind. Eng. Chem. Fundam..

[67] Hameed B (2009). Evaluation of papaya seeds as a novel non-conventional low-cost adsorbent for removal of methylene blue. J. Hazard. Mater..

[68] Chanzu HA, Onyari JM, Shiundu PM (2012). Biosorption of malachite green from aqueous solutions onto polylactide/spent brewery grains films: kinetic and equilibrium studies. J. Polym. Environ..

[69] Özcan AS, Erdem B, Özcan A (2005). Adsorption of acid blue 193 from aqueous solutions onto BTMA-bentonite. Colloids Surf. A.

[70] Yadav S, Srivastava V, Banerjee S, Weng C-H, Sharma YC (2013). Adsorption characteristics of modified sand for the removal of hexavalent chromium ions from aqueous solutions: Kinetic, thermodynamic and equilibrium studies. CATENA.

[71] Bayramoğlu G, Ozalp VC, Arıca MY (2017). Removal of Disperse Red 60 dye from aqueous solution using free and composite fungal biomass of *Lentinus concinnus*. Water Sci. Technol..

[72] AbdEl-Mageed H, Mustafa F, Abdel-Latif MK (2020). The ability of gold nanoclusters as a new nanocarrier for D-penicillamine anticancer drug: a computational chemistry study. Struct. Chem..

