# Author Correction: Experimentally and theoretically approaches for disperse red 60 dye adsorption on novel quaternary nanocomposites

**DOI:** 10.1038/s41598-021-96498-y

**Published:** 2021-08-17

**Authors:** N. K. Soliman, A. F. Moustafa, H. R. Abd El-Mageed, Omima F. Abdel-Gawad, Esraa T. Elkady, Sayed A. Ahmed, Hussein S. Mohamed

**Affiliations:** 1 Basic Science Department, Nahda University, Beni-Suef, Egypt; 2 Ministry of Health and Population, Central Administration of Environmental Affairs, Beni-Suef Branch, Beni-Suef, Beni-Suef Governorate Egypt; 3 Faculty of Science, Micro-Analysis and Environmental Research and Community Services Center, Beni-Suef University, Beni-Suef City, Egypt; 4 Chemistry Department, Faculty of Science, Beni-Suef University, Beni-Suef City, Egypt; 5 Research Institute of Medicinal and Aromatic Plants (RIMAP), Beni-Suef University, Beni-Suef City, Egypt

Correction to: *Scientific Reports* 10.1038/s41598-021-89351-9, published online 11 May 2021.

The original version of this Article contained an error in the spelling of the author H. R. Abd El-Mageed which was incorrectly given as H. R. Abdel El-Mageed. The original Article and accompanying Supplementary Information file have been corrected.

