# Supplementary material for: Experimentally and theoretically approaches for disperse red 60 dye adsorption on novel quaternary nanocomposites
Source: Sci Rep. 2021 May 11;11:10000. doi: 10.1038/s41598-021-89351-9 (PMC8113254; doi:10.1038/s41598-021-89351-9)
Supplement: Supplementary file 1 — Supplementary Information. [file 41598_2021_89351_MOESM1_ESM.docx]

**Experimentally and theoretically approaches** **for disperse red 60 dye adsorption on novel quaternary nanocomposites**

N. K. Soliman^a,*^, A. F. Moustafa^b^, H. R. Abd El-Mageed^c^, Omima F. Abdel-Gawad^d^, Esraa T.Elkady^d^, Sayed A. Ahmed^d^, Hussein S. Mohamed^e^.

a) Basic science department, Nahda University, Beni-Suef, Egypt.

b) Ministry of Health and Population, Central Administration of Environmental Affairs

Beni-Suef Branch, Beni-Suef governorate, EGYPT.

c) Micro analytical lab, Faculty of Science, Beni-Suef University, Egypt.

d) Chemistry department, Faculty of Science, Beni-Suef University, Egypt.

e) Research Institute of Medicinal and Aromatic Plants (RIMAP), Beni-Suef University, Egypt.

*Corresponding Author: Nofal khamis (Email: [nofal.khamis@nub.edu.eg](mailto:nofal.khamis@nub.edu.eg))

Table.S1; FTIR data of chitosan, its Schiff base derivatives and the novel synthesized adsorbents

| Ce-O | Fe-O | Cu-O | Al-O | OH  Bending | C-C  Aromatic  Ring | C-H  Stretch | C-N | C=N  Stretch | NH_2_ | OH Stretch | Types of vibration        Types of  Derivatives |
| --- | --- | --- | --- | --- | --- | --- | --- | --- | --- | --- | --- |
| Peaks position (cm^-1^) | | | | | | | | | | |  |
| 903 |  |  | 1097 | 1648 |  |  |  |  |  |  | CuO-CeO_2_-Al_2_O_3_ |
| 882 | 425 | 598 |  | 1674 |  |  |  |  |  |  | CuO-CeO_2_-Fe_2_O_3_ |
|  |  |  |  |  | - | 2914 | 1347 | - | 3133 | 3241 | Chitosan |
|  |  |  |  |  | 1295 | 2957 | 1286 | 1657 | - | 3336 | Chitosan-4-chloroacetoPhenone |
| 968 |  | 589 | 1080 | 1631 |  |  |  |  |  |  | CF before adsorption |
| 942 |  | 572 | 1045 | 1640 |  |  |  |  |  |  | CF after adsorption |
| 916 | 494 | 649 |  | 1675 |  |  |  |  |  |  | CA before adsorption |
| 882 | 483 | 598 |  | 1657 |  |  |  |  |  |  | CA after adsorption |

Table.S2; TGA data of chitosan and its Schiff base derivatives

| **Total Wt loss %** | **Samples Wt loss % at** | | | | |
| --- | --- | --- | --- | --- | --- |
|  | 565^o^C | 430^o^C | 320^o^C | 100^o^C |  |
| **95** | 30 | 8 | 47 | 10 | Chitosan |
| **100** | 30 | 13 | 47 | 10 | Chitosan-4-chloroacetophenone |

| **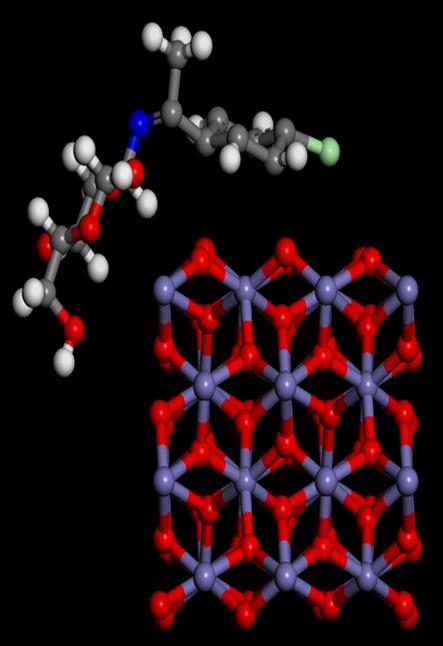**  **(CF-1nm )** | **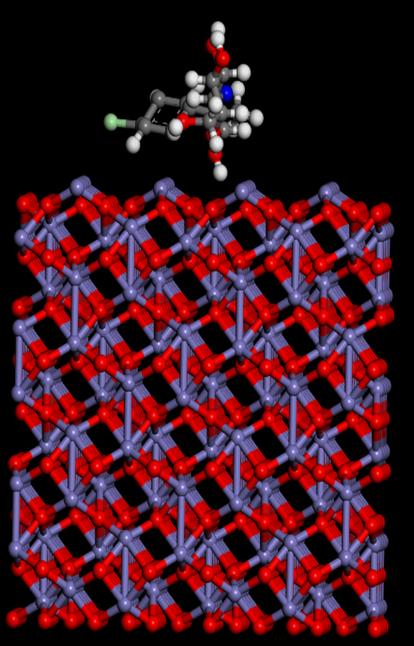**  **(CF-2nm )** | **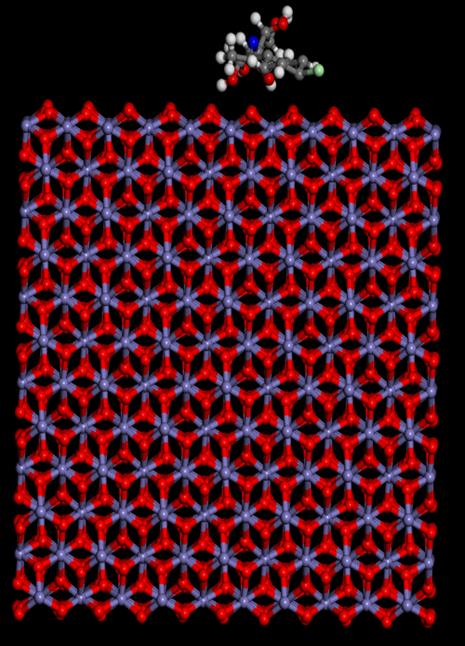**  **(CF-3nm )** |
| --- | --- | --- |

**Figure S1: The lowest configurations obtained due to the interactions between chitosan-4-chloroacetophenone Schiff base with Fe_2_O_3_ (CF) for different size (1,2, and 3nm)**

| **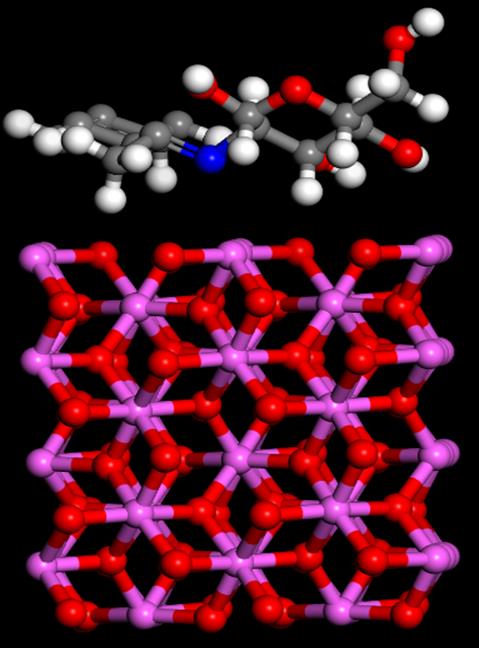**  **(CA-1nm )** | **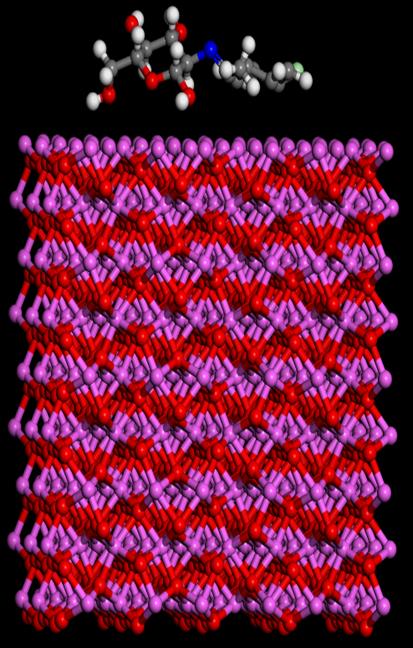**  **(CA-2nm )** | **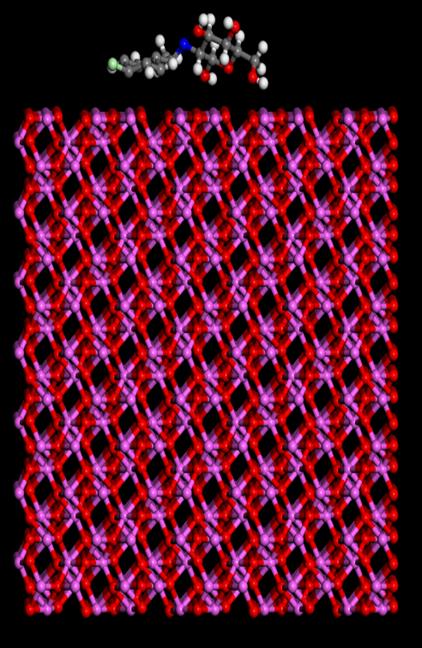**  **(CA-3nm )** |  |
| --- | --- | --- | --- |

**Figure S2: The lowest configurations obtained due to the interactions between chitosan-4-chloroacetophenone Schiff base with Al_2_O_3_ (CA) for different size (1,2, and 3nm)**

| **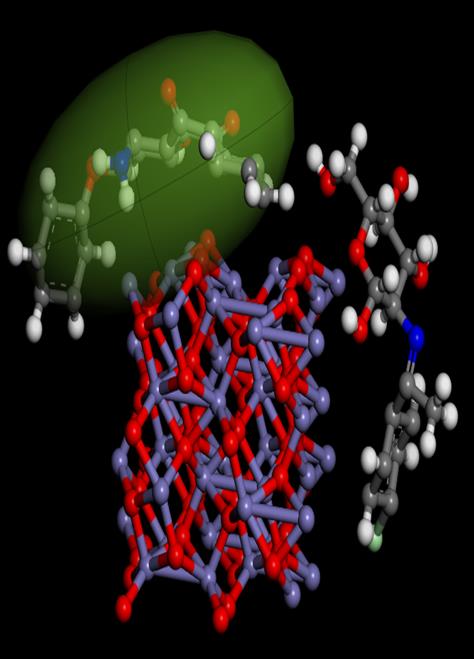**  **DR-(CF-1nm )** | **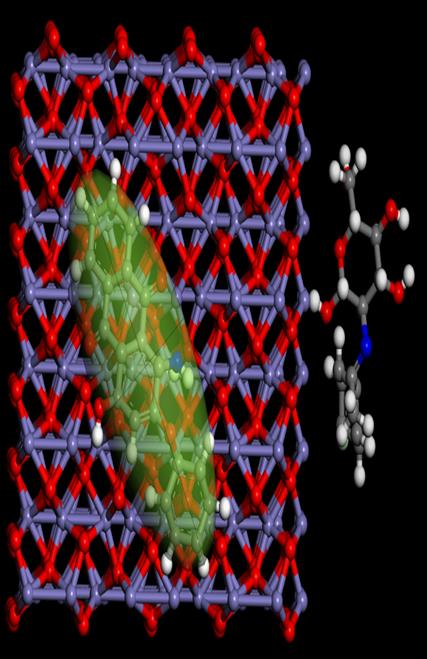**  **DR-(CF-2nm )** | **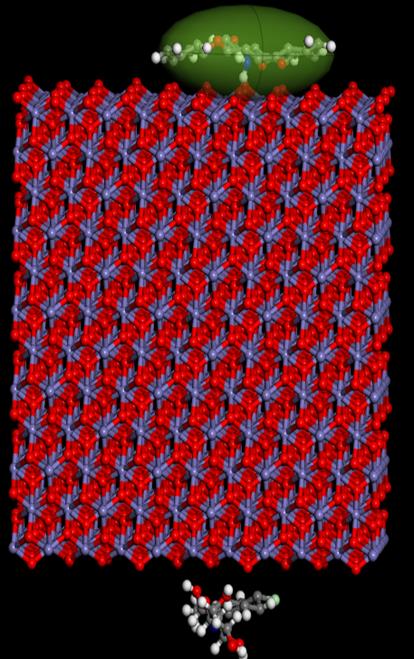**  **DR-(CF-3nm )** |
| --- | --- | --- |

**Figure S3: The adsorption configurations of DR adsorbed on CF-simple box, at different size (1,2, and 3nm); DR, displayed in ellipsoid structure for better showing.**

| **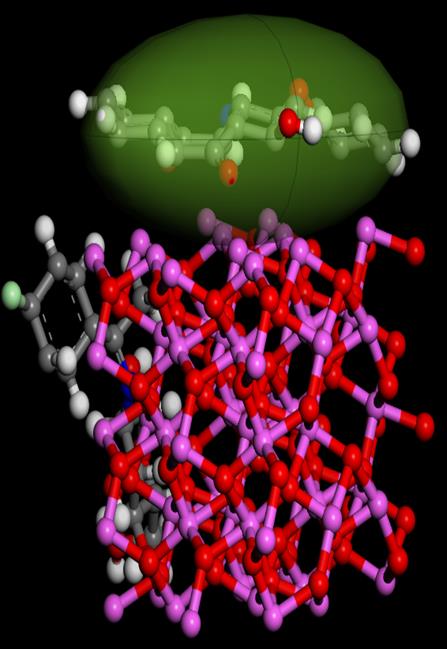**  **DR-(CA-1nm )** | **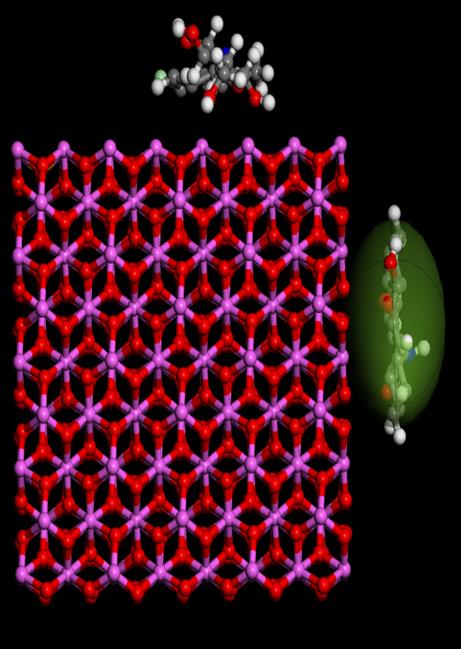**  **DR-(CA-2nm )** | **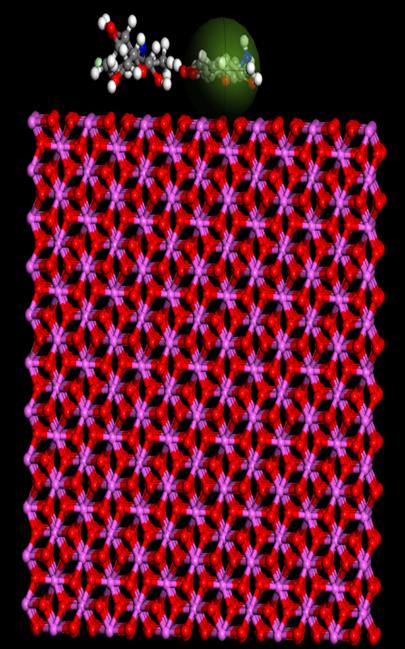**  **DR-(CA-3nm )** |
| --- | --- | --- |

**Figure S4: The adsorption configurations of DR adsorbed on CA simple box, at different size (1,2, and 3nm); DR, displayed in ellipsoid structure for better showing.**
